# Carbon storage in Chinese grassland ecosystems: Influence of different integrative methods

**DOI:** 10.1038/srep21378

**Published:** 2016-02-17

**Authors:** Anna Ma, Nianpeng He, Guirui Yu, Ding Wen, Shunlei Peng

**Affiliations:** 1 Key Laboratory of Ecosystem Network Observation and Modeling, Institute of Geographic Sciences and Natural Resources Research, Chinese Academy of Sciences, Beijing, 100101, China

## Abstract

The accurate estimate of grassland carbon (C) is affected by many factors at the large scale. Here, we used six methods (three spatial interpolation methods and three grassland classification methods) to estimate C storage of Chinese grasslands based on published data from 2004 to 2014, and assessed the uncertainty resulting from different integrative methods. The uncertainty (coefficient of variation, CV, %) of grassland C storage was approximately 4.8% for the six methods tested, which was mainly determined by soil C storage. C density and C storage to the soil layer depth of 100 cm were estimated to be 8.46 ± 0.41 kg C m^−2^ and 30.98 ± 1.25 Pg C, respectively. Ecosystem C storage was composed of 0.23 ± 0.01 (0.7%) above-ground biomass, 1.38 ± 0.14 (4.5%) below-ground biomass, and 29.37 ± 1.2 (94.8%) Pg C in the 0–100 cm soil layer. Carbon storage calculated by the grassland classification methods (18 grassland types) was closer to the mean value than those calculated by the spatial interpolation methods. Differences in integrative methods may partially explain the high uncertainty in C storage estimates in different studies. This first evaluation demonstrates the importance of multi-methodological approaches to accurately estimate C storage in large-scale terrestrial ecosystems.

Grasslands store approximately 10% of the organic carbon (C) in global terrestrial ecosystems^1^. It has been estimated that C storage in Chinese grasslands accounts for 8% of C storage in global grasslands^2^ and 16.7% of C storage in Chinese terrestrial ecosystems^3^. Some studies have demonstrated that Chinese grasslands have tremendous potential for increasing C storage through improved grassland use or management^4^,^5^,^6^,^7^,^8^. Enhancing grassland C storage is currently considered one of the most effective and economical approaches to partially sequester anthropogenic CO_2_ emissions in China^9^,^10^,^11^. Therefore, how to accurately assess C storage in Chinese grasslands is of concern for both scientists and policymakers.

Some studies have estimated C storage of different components in Chinese grasslands using various data source. Fang *et al.*^10^ and Ni^12^ estimated the C storage of above-ground biomass (AGB) based on Grassland Resource Survey data. Yang *et al.*^13^ estimated AGB in northern China’s grasslands by field-investigated data. Some other researchers estimated the C storage of below-ground biomass (BGB) using empirical data based on root to shoot ratios (R/S)^3^,^14^,^15^. However, current estimates of C storage in different grassland components are subject to high variation. For instance, estimates of vegetation C density range from 0.22 kg C m^−2 ^3 to 1.15 kg C m^−2 ^2, and estimates of C storage range from 0.56 Pg C^16^ to 4.66 Pg C^2^ in vegetation. Similarly, estimates of soil organic C (SOC) storage range from 16.70 Pg C^16^ to 53.72 Pg C^2^. The large difference among these estimates may be resulted from the difference in data used for the estimation. In addition, the application of the different methods would be another factor affecting the estimates.

Different methods have been used to estimate C storage in the different components of Chinese grasslands, including remote sensing^14^, modeling^16^, and field investigation data^13^. However, soil C estimate was insufficient in Chinese grasslands because the remote sensing method was not appropriate to estimate it. Based on this, field-investigated data were always used to estimate C storage, especially soil C, at the national scale by the mean C density method. Considering the field-investigate data was scarce, the data has been integrated according to grassland type^12^, climate zone^13^, administrative region^14^, and spatial interpolation^17^. Therefore, differences in the various scale-up methods maybe have primarily contributed to the inconsistency of results among different studies. To the best of our knowledge, no study has attempted to investigate how different integrative methods influence the estimation of C storage of all main components (AGB, BGB, and SOC) in Chinese grasslands using field-measured data at the national scale and the associated uncertainty.

In this study, we collected field-measured data from papers published from 2004 to 2014, including AGB, BGB, and SOC, and used six integrative methods (three spatial interpolation methods and three grassland classification methods) to calculate the C storage of different components in Chinese grasslands. We have addressed two fundamental questions in this paper: (1) To investigate the uncertainty of the estimates of C density and storage in grasslands resulting from the selected integrative methods, and (2) To evaluate C storage in all components of Chinese grasslands (including AGB, BGB, and SOC) on the basis of different integrative methods at the national scale.

## Results

### Carbon density and storage based on spatial interpolation methods

**Figure 1:**
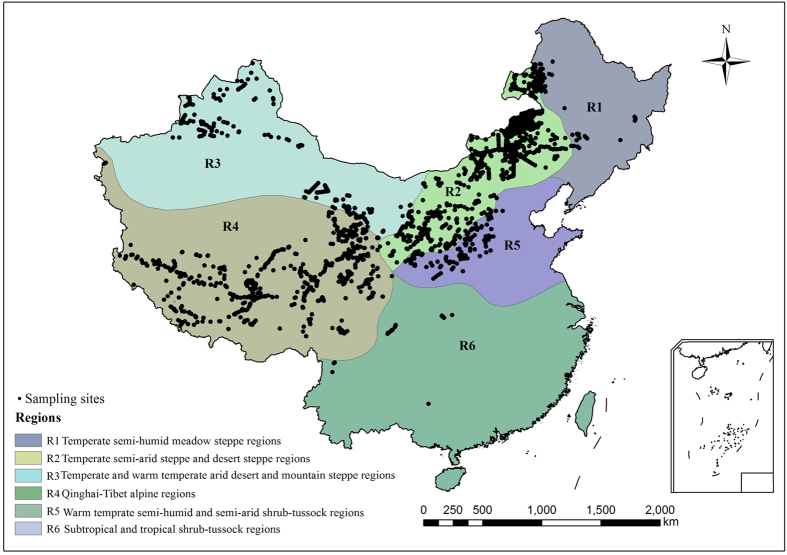
Locations of the sampling sites and ecological regions. The figure was created using Arcgis 8.2 software (ESRI Inc., Redlands, CA).

**Figure 2:**
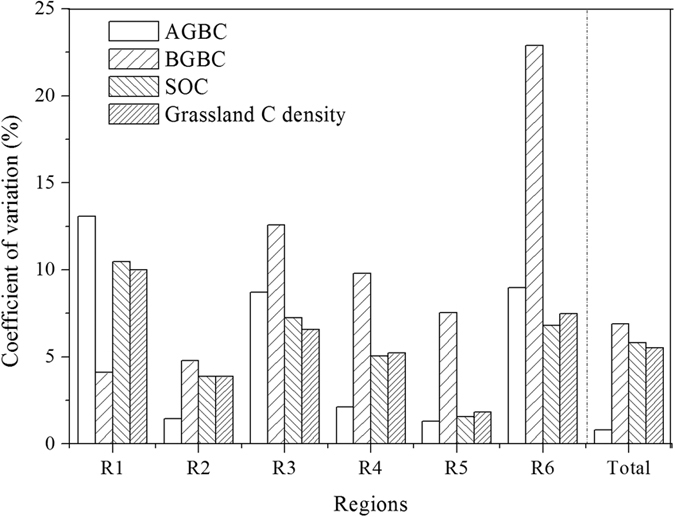
Coefficient of variation (CV, %) of carbon density for three spatial interpolation methods applied to different components and regions. AGBC, carbon density of the above-ground biomass; BGBC, carbon density of the below-ground biomass; SOC, SOC density. R1, temperate semi-humid meadow steppe regions; R2, temperate semi-arid steppe and desert steppe regions; R3, temperate and warm temperate arid desert and mountain steppe regions; R4, Qinghai-Tibet alpine regions; R5, warm temperate semi-humid and semi-arid shrub-tussock regions; R6, subtropical and tropical shrub-tussock regions.

The uncertainty (coefficient of variation [CV], %) of C density estimated by three spatial interpolation methods was different among six grassland regions (Fig. 1) and different components (Fig. 2). The range of AGBC was 0.018 ~ 0.312 kg C m^−2^, and the highest and lowest CVs for AGBC were in R1 (13.1%) and R5 (1.3%). The range of BGBC was 0.061 ~ 1.314 kg C m^−2^, and the highest and the lowest CVs for BGBC were in R6 (22.9%) and R1 (4.1%). For SOC, R1 and R3 had higher CVs, which were 10.5% and 7.3%, respectively. The range of SOC was 1.892 ~ 24.856 kg C m^−2^, and the CV value was about 5.8%. Overall, the CV values of C density were highest in BGBC, followed by SOC and AGBC (Fig. 2).

Furthermore, the CVs of grassland C density differed among regions, with a mean CV of approximately 5.5% (Fig. 2). The CV value of grassland C density was close to the variation of SOC density, because 94.8% of C was found to be stored in the soils of Chinese grasslands.

**Figure 3:**
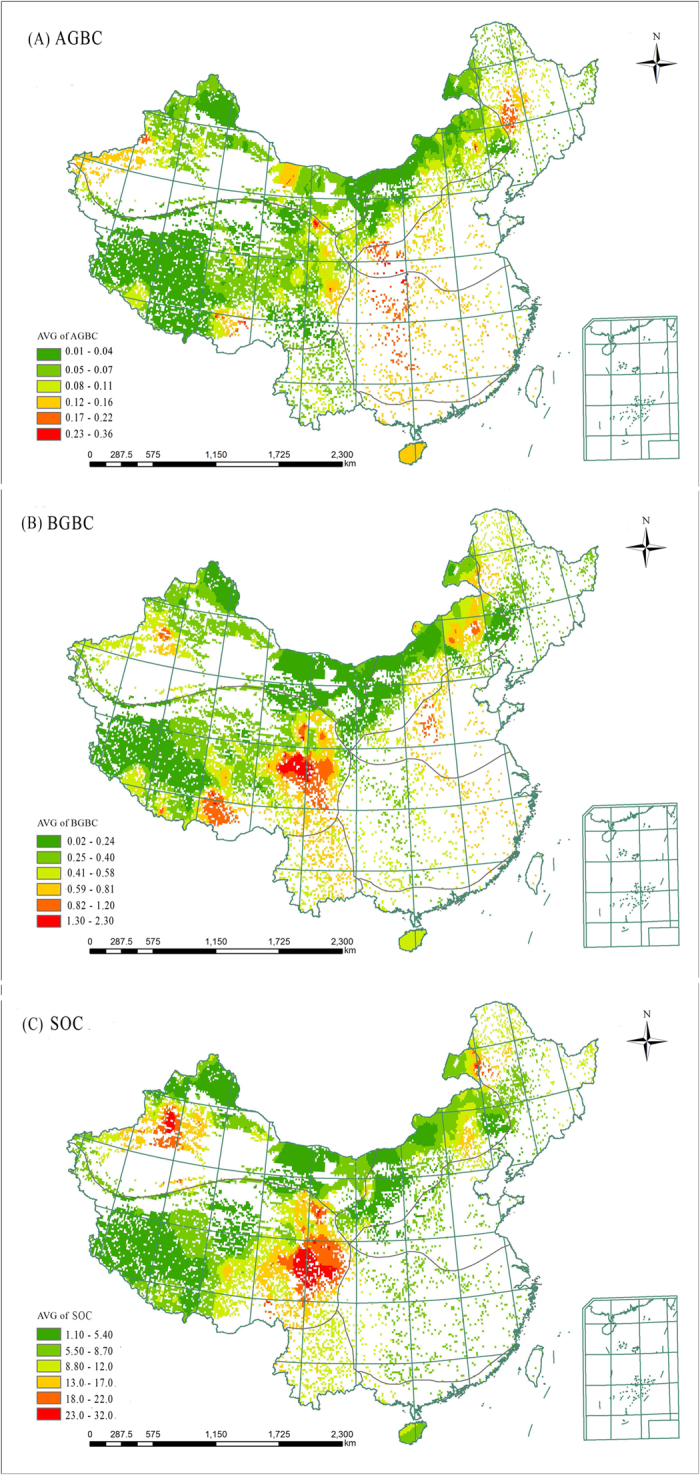
Spatial pattern of carbon density (kg C m^−2^) in the above-ground biomass (AGBC, **A**), below-ground biomass (BGBC, **B**), and soil (SOC, **C**). The figure was created using Arcgis 8.2 software (ESRI Inc., Redlands, CA).

The C density of different components in Chinese grasslands differed significantly in different regions (F = 62.10, *P* < 0.01 for AGBC; F = 11.46, *P* < 0.01 for BGBC; F = 13.25, *P* < 0.01 for SOC; F = 11.99, *P* < 0.01 for grassland C density; Table 1). The C density of Chinese grasslands at the national scale was estimated as 8.66 ± 0.50 kg C m^−2^ using three spatial interpolation methods, which estimated AGBC, BGBC, and SOC as 0.08 ± 0.01, 0.42 ± 0.03, and 8.16 ± 0.48 kg C m^−2^, respectively (Fig. 3). Overall, the total C storage in Chinese grasslands (including AGB, BGB, and SOC in the 0–100 cm soil layer) was 31.49 ± 1.74 Pg C.

### Carbon density and storage estimates using grassland classification methods

**Figure 4:**
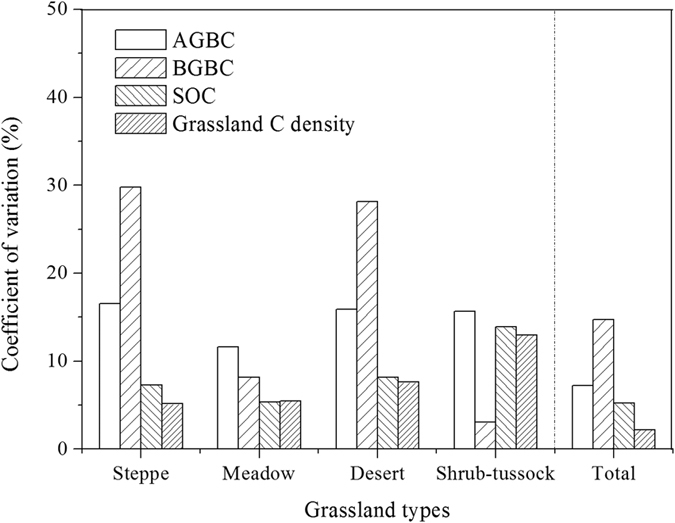
Coefficient of variation (CV, %) of carbon density on three grassland classification scales in different components and grassland types. AGBC, carbon density of the above-ground biomass; BGBC, carbon density of the below-ground biomass; SOC, SOC density.

The CV values resulting from different grassland classification methods were different for the different components in Chinese grasslands (Fig. 4). The CV value for AGBC exceeded 10% in all grassland types, and the CV value of all grasslands was 7.2%, which ranged 0.021 to 0.342 kg C m^−2^. The CV of BGBC for all grasslands 9.8%, with higher values found in steppe (29.8%) and in desert (28.2%), and the range of BGBC was 0.041 ~ 1.844 kg C m^−2^. The CV value of SOC in all grassland types was 5.2%, with the highest CV in shrub-tussock (13.9%), and the range of SOC was 2.197 ~ 21.850 kg C m^−2^. The CV values of grassland C density ranged from 5.2% to 13.0% across all grassland types, with a mean CV of 2.2%.

The C density differed significantly in the different components of various grassland types (F = 29.90, *P* < 0.01 for AGBC; F = 60.49, *P* < 0.01 for BGBC, F = 46.32, *P* < 0.01 for SOC, and F = 50.55, *P* < 0.01 for grassland; Table 2). The mean C densities for AGB, BGB, SOC, and grassland were 0.08 ± 0.01, 0.41 ± 0.04, 7.76 ± 0.16, and 8.25 ± 0.18 kg C m^−2^, respectively. Correspondingly, total C storage was estimated as 30.47 ± 0.28 Pg C for Chinese grasslands (down to 100 cm soil layer), with AGB, BGB, and SOC containing 0.24 ± 0.02, 1.39 ± 0.21, and 28.84 ± 0.14 Pg C, respectively.

### Carbon storage in Chinese grasslands and uncertainty

**Figure 5:**
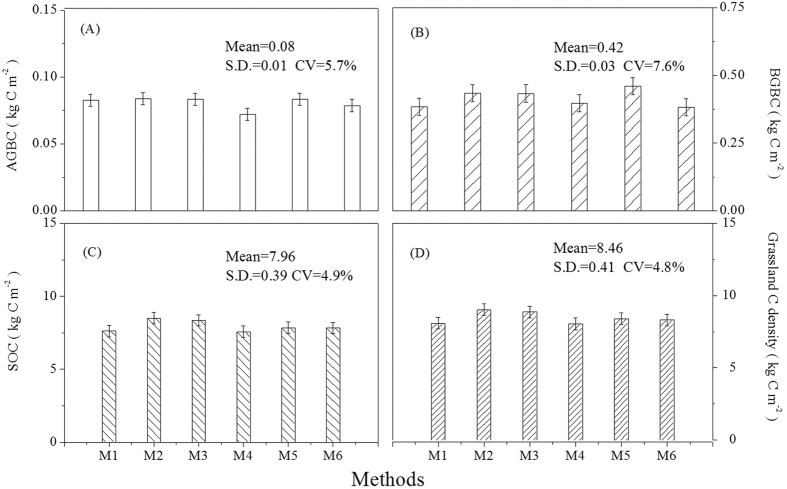
Comparison of carbon density estimated from the six integrative methods. (**A**) carbon density of the above-ground biomass (AGBC); (**B**) carbon density of the below-ground biomass (BGBC); (**C**) SOC density (SOC); (**D**) grassland carbon density. See Table 4 for methodological descriptions.

Figure 5 provides the C density estimates for different components in Chinese grasslands based on six integrative methods. The mean C densities of AGB, BGB, and SOC were 0.08 ± 0.01, 0.42 ± 0.03, and 7.96 ± 0.39 kg C m^−2^, respectively, at the national scale. Overall, the C density of BGB had the highest CV (7.6%) among all components. However, the CV value of grassland C density was close to that of SOC, where they were 4.8% and 4.9%, respectively. The C density of Chinese grasslands was 8.46 ± 0.41 kg C m^−2^, most of which stored as SOC.

**Figure 6:**
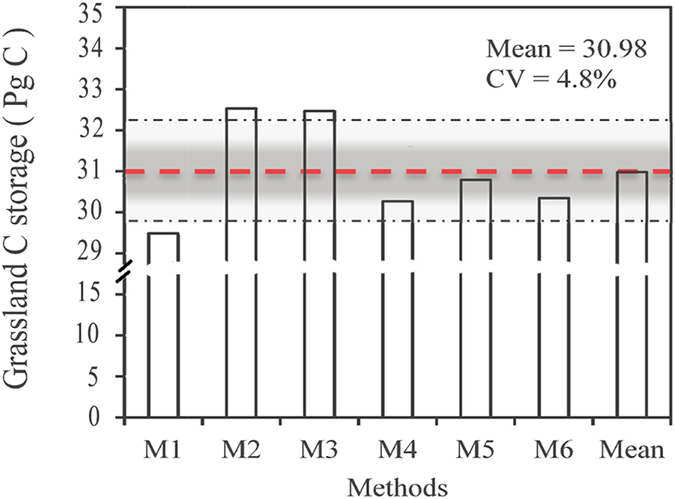
Changes in the estimation of carbon storage in Chinese grasslands for six integrative methods. The methods are described in Table 4. Red line indicates the mean value of the six methods, and the rectangular area is the 95% confidence interval.

When combining all grassland areas, the C storage of grassland (including AGB, BGB, and SOC down to the 100 cm soil layer), estimated by the six integrative methods, ranged from 29.49 to 32.53 Pg C, with a mean of 30.98 Pg C (Fig. 6). The C storage in Chinese grasslands was composed of 0.7% AGBC (0.23 ± 0.01 Pg C), 4.5% BGBC (1.38 ± 0.14 Pg C), and 94.8% SOC (29.37 ± 1.20 Pg C). In general, the estimates obtained from the three spatial interpolation methods tended to be higher than those from grassland classification methods. The estimate of M5, which classified Chinese grasslands into 18 types, was closest to the average of grassland C storage calculated by the six methods (30.79 Pg C).

## Discussion

This study provided the first assessment of the C density and C storage of all main components in Chinese grasslands at the national scale using the six integrative methods. The results found that uncertainty of C storage estimation in Chinese grasslands, resulting from the selected scale-up methods, was approximately 4.8%. However, the level of uncertainty appeared to vary among the different components of grasslands, with the highest and lowest uncertainty being obtained from BGBC and SOC, respectively. One reason for the high uncertainty of BGBC may be the data distribution of BGBC, as the values varied from 0.002 kg C m^−2^ to 3.44 kg C m^−2^. Although expanding the soil depth to 100 cm may result in overestimation of BGBC to some extent, the influence on the ecosystem C estimates was not noticeable because of the small contribution of BGBC in deeper soil layers to total BGBC (Supplementary Fig. S1) and the small proportion of vegetation C density in grasslands.

SOC accounted for 94.8% of grassland C in this study, which was similar to those of previous studies that found that soil stores 90–97% of grassland C^4^,^18^,^19^. Considering the importance of SOC for grassland C estimates, regional variation (CV) of the C density of grasslands mainly depended on SOC variation. Up to date, it is impossible to accurately estimate soil C storage in Chinese grasslands by remote-sensing methods (or satellite-based approach). In order to keep the consistency of methods to estimate the C densities of AGBC, BGBC, and SOC, the methods of spatial-interpolation and mean density therefore were used to estimate vegetation C storage in this study, although the linkage between field investigation and remote-sensing should improve the estimate of AGBC, to some extent.

The CV values of grassland C density calculated from the three spatial interpolation methods (5.5%) were higher than those calculated from the three grassland classification methods (2.2%). Our results demonstrated that the spatial interpolation methods have higher uncertainty of estimates, because they were more easily affected by the data quantity and spatial distribution of sampling sites^20^. Furthermore, the spatial interpolation methods generally produced higher estimates (8.66 ± 0.50 kg C m^−2^) of grassland C density than the grassland classification methods (8.25 ± 0.18 kg C m^−2^). One possible explanation is that the spatial interpolation methods may be affected more by the number of sampled sites and nearby sites, particularly those with higher C density. The grassland C estimate was mainly decided by SOC. The SOC estimate in the 0–100 cm soil layer obtained using the six integrative methods was 7.96 kg C m^−2^ in this study, which was similar to the previous results of (7.80 kg C m^−2^) Yang *et al.*^23^ and (8.50 kg C m^−2^) Fang *et al.*^18^. The SOC estimate here was 3.33 kg C m^−2^ in the 0–20 cm soil layer, which was about 41.8% of that in the 0–100 cm and was similar to the average value of global grasslands (42%)^24^. For all methods, the estimation of grassland C storage using 18 vegetation types produced the value closest to the mean of the six methods, and this method was used by Fan *et al.*^21^ and Ni^22^. This result indicates that integrative methods require a suitable scale to ensure that sampling sites in a given region are sufficient, particularly when the field-measured sites are uncertain. These findings provide new insights showing that multiple approaches, especially the multiple-scale integrative method, should be used to estimate the C storage in terrestrial ecosystem at large scales.

The estimation of grassland C storage at the national scale was limited and was apparently different among the previous studies (Table 3).One possible explanation is the different data sources for C density^10^. Previous studies mainly used data from the Grassland Resource Survey in China to estimate the C density of AGB^10^,^13^,^14^,^25^ (Table 3), and BGB was deduced from AGB using R/S ratios^13^,^14^, whereas others used the global mean vegetation C density to estimate vegetation C density^2^,^22^. The global mean soil C density^2^,^22^ or data from the Second Soil Survey in China^26^ were used to estimate SOC density in Chinese grasslands. Furthermore, Yang *et al.*^16^,^17^ used field-measured data to estimate the C density of vegetation and SOC in northern China’s grasslands, and found that the C density of AGB, BGB, and SOC was approximately 0.04, 0.22, and 8.49 kg C m^−2^, respectively. In addition, Yang *et al.*^27^ estimated Chinese grassland vegetation C storage and SOC storage to be 0.90 Pg C and 35.06 Pg C, respectively, by remote sensing data. Different data sources resulted in different estimates. Therefore, we concluded that these major differences in data sources are important reasons for the high uncertainty of C storage in Chinese grasslands.

A second possible explanation for these differences is the lack of data for key components in grasslands, especially synchronous measurements. Vegetation C density could be estimated by many methods, such as remote sensing, modelling, and field-investigation. However, SOC is difficult to be estimated by remote sensing and modelling, although SOC is the most important component in grassland ecosystems. As shown in Table 3, the calculation of C density in AGB was 0.06 ± 0.03 kg C m^−2^ with 47.4% variation, whereas the C density in BGB was 0.41 ± 0.27 kg C m^−2^ with 66.4% variation. It was estimated that the C density of BGB was approximately 7 times higher than that of AGB, which was consistent with previous results suggesting that 86–88% of vegetation C density is stored in BGB^21^,^28^. However, there are few reports of *in situ* measurements of BGB, despite such measurement being important for the estimation of vegetation C density. The R/S ratio has been verified as a good parameter for inferring BGB at the regional scale; however, this measure also generates uncertainty due to various disturbances (e.g., grazing, mowing). Furthermore, previous studies using global mean soil C density^2^,^22^ overestimated the C storage in Chinese grasslands (Table 3). Therefore, the high variation in C storage estimates derived from the different components of grasslands among different researches mainly resulted from different or nonsynchronous measurements.

Additionally, this study provided the first estimate of three components by field-measurement data and demonstrated that integrative methods have an important influence on the estimation of grassland C storage at the national scale. However, previous studies only used one method to estimate one or two components. Ni^12^ used the Grassland Resource Survey to estimate vegetation C density based on grassland classification integrative methods. In comparison, Piao *et al.*^14^ used the same data source to estimate C density based on modeling. Their estimates of AGB in Chinese grasslands were 0.06 kg C m^−2^ and 0.04 kg C m^−2^. Therefore, multiple approaches should be used to estimate the C storage in terrestrial ecosystems and improve the accuracy of C storage estimates at large scales. The uncertainty of C storage in Chinese grasslands using the six integrative methods was approximately 4.8%. The level of uncertainty differed among different components, with the highest values observed in BGBC. Our findings emphasize the underlying influence of integrative methods for estimating C storage in terrestrial ecosystems at a large scale. Based on the six integrative methods, C storage in Chinese grasslands was estimated as 0.23 ± 0.01, 1.38 ± 0.14, 29.37 ± 1.2, and 30.98 ± 1.25 Pg C in AGBC, BGBC, SOC, and grasslands, respectively. This first assessment of C storage in all main components of Chinese grasslands at the national scale will help to determine the potential roles of Chinese grasslands in response to global climate change.

## Methods

### Data sources

C density data for different components of Chinese grasslands were derived from: (1) field-measured data of 210 papers publicly published from 2004 to 2014 in the Web of Science (www. Webofknowledge.com) and in the China National Knowledge Infrastructure (http://epub.cnki.net) (Supplementary Appx. S1 ~ S4), in addition to (2) some unpublished field-measured data obtained by personal correspondence (Supplementary Appx. S1).

Furthermore, we obtained the Chinese grassland areas and the spatial distribution of different grassland types from the map of grassland resources in China at 1:4M^29^. Based on this map, the total area of Chinese grasslands was estimated as 3.55 × 10^8^ ha.

### Data processing

Field-measured above-ground biomass (AGB, kg m^−2^) records were used directly. If the AGB data were measured monthly in the published papers, we chose the values measured in August, which is commonly considered to be the peak period for AGB in Chinese grasslands^4^. The C content of AGB and below-ground biomass (BGB, kg m^−2^) in grasslands was estimated to comprise 45% of the plant dry matter^3^; thus, we calculated the C density of AGB (AGBC, kg C m^−2^) from AGB and the coefficient of C content (0.45).

Similarly, the C density of BGB (BGBC, kg C m^−2^) was calculated from BGB and the coefficient of C content (0.45). In practice, the BGB data were directly used if the sampling depth was 100 cm. For data obtained at a soil depth of less than 100 cm, we estimated the BGBC as a 0–100 cm soil layer based on the 0–20 cm data using the following procedure. We first established the relationships of BGBC between 0–20 cm and 0–100 cm using 99 sampling sites where data were simultaneously collected at 20 cm and 100 cm depths. We found that BGBC showed a significantly positive correlation between 0–20 cm and 0–100 cm (R^2^ = 0.97, *P* < 0.001; Supplementary Fig. S1). Therefore, the BGBC values in the 0–100 cm soil layer may be deduced from surface soil (0–20 cm) data by the following formula:


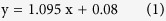


where y and x are BGBC at the 100 cm and 20 cm depths, respectively.

SOC density (SOC, kg C m^−2^) was calculated using equation 2, unless SOC data were reported directly in the published papers. In practice, we commonly used the field-measured bulk density. For soil samples without bulk density records, we used the mean values of bulk density from the second soil survey in China (1.3 g cm^−3^) as a substitute 30. For soil samples from a depth of less than 100 cm, we adopted the empirical relationships between soil C content and depth to fit to the 100 cm soil layer. In a previous paper, we established the empirical relationships in the 74 terrestrial ecosystems of China using the long-term monitoring data of the Chinese Ecosystem Research Network^31^. Here, we randomly selected 118 sites to validate the accuracy of the prediction, and found that the predicted SOC values are closely correlated and almost equal to the measured values in the 0–100 cm soil layer (y = 1.08 × –0.19, R^2^ = 0.95) (Supplementary Fig. S2).


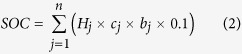


where SOC is SOC density (kg C m^−2^), *H*_*j*_ is soil thickness (cm), *c*_*j*_ is the concentration of SOC (%), *b*_*j*_ is bulk density (g cm^−3^).

Overall, the total number of sampling sites for AGBC, BGBC, and SOC was 2659, 1022, and 991, respectively. The distribution of the sampling sites is shown in Fig. 1.

### Integrative methods

To assess the influence of different integrative methods on C storage estimates in the scaling-up process in this study, we adopted three spatial interpolation methods and three grassland classification methods (Table 4).

### Spatial interpolation methods

The geo-statistical principle assumes that grasslands gradually change with climate. Theoretically, the biomass at one sampling site may have the highest similarity to that at the nearest site. Based on this assumption, spatial interpolation methods may be used to estimate the C storage of grasslands in China. In practice, we selected three spatial interpolation methods, *viz.* Kriging interpolation (M1), Inverse Distance Weighted interpolation (M2), and Empirical Bayesian Kriging interpolation (M3). Among these methods, M2 only considers the distance, whereas M1 and M3 consider both the spatial orientation and the distance.

For statistical and comparative analyses, the grasslands were divided into six regions according to the nationwide grassland resource survey of DAHV & GSAHV^25^, which were designated as temperate semi-humid meadow steppe regions (R1), temperate semi-arid steppe and desert steppe regions (R2), temperate and warm temperate arid desert and mountain steppe regions (R3), Qinghai-Tibet alpine regions (R4), warm temperate semi-humid and semi-arid shrub-tussock regions (R5), and subtropical and tropical shrub-tussock regions (R6) (Fig. 1).

### Grassland classification methods

Grasslands exhibit different ecological characteristics based on climate (temperature and precipitation), topography, and soil. Therefore, grasslands may be divided into different artificial types. Based on this assumption, C storage in Chinese grasslands may be calculated from grassland type and the corresponding area at different scales (Table 4 and Supplementary Appx. S5). The major aim is to explore C storage at the national scale based on the C density of different grassland types.

For the statistical and comparative analyses, the Chinese grasslands were classified at three different scales. First, 5 grassland types of China’s vegetation were determined at 1:1000000 resolution^32^ (M4): steppe, meadow, desert, shrub-tussock, and swamp. Swamps were excluded in this study because of their small area and insufficient sampling sites. Second, 18 grassland types based on the national grassland survey were used^25^ (M5) (Supplementary Appx. S5). Third, 32 grassland subtypes based on the national grassland survey were used^25^ (M6).

### Statistical analysis

The coefficient of variation (CV, %) was used to assess the uncertainty of the six methods for estimating C storage^33^,^34^, which was defined as the ratio of the standard deviation to the mean. The range of of AGBC, BGBC and SOC came from the maximal and the minimum value, and the median value calculated by different methods was used. Grassland C storage was summarized from the C storages in AGB, BGB, and SOC in the 0–100 cm soil layer. One-way variance analysis (ANOVA) was used to test for differences in C density in different regions or grassland types. Spatial interpolation and statistical analysis were performed by Arcgis 8.2 (ESRI Inc., Redlands, CA) and SPSS 13 software. Significant differences were defined as *P* = 0.05.

## Additional Information

**How to cite this article**: Ma, A. *et al.* Carbon storage in Chinese grassland ecosystems: Influence of different integrative methods. *Sci. Rep.* **6**, 21378; doi: 10.1038/srep21378 (2016).

## Supplementary Material

Supplementary Information

Supplementary datasets 1

## Figures and Tables

**Table 1:** Regional distribution and variation in carbon density and storage of Chinese grasslands using three spatial interpolation methods.

Region	Area(10^6^ha)	C density (kg C m^−2^)					C storage (Pg C)			CV
		AGBC§	BGBC	SOC	Ecosystem	AGBC	BGBC	SOC	Ecosystem	%
R1‡	19.2	0.094^a†^	0.369^ab^	9.359^a^	9.823^a^	0.018	0.071	1.794	1.883	10.0
		(0.012)	(0.015)	(0.979)	(0.981)	(0.002)	(0.003)	(0.188)	(0.188)	
R2	63.6	0.059^b^	0.345^ab^	6.944^bc^	7.348^b^	0.037	0.219	4.415	4.672	3.9
		(0.001)	(0.016)	(0.268)	(0.284)	(0.001)	(0.010)	(0.170)	(0.180)	
R3	76.8	0.062^b^	0.285^a^	7.792^b^	8.139^b^	0.048	0.219	5.984	6.250	6.6
		(0.005)	(0.036)	(0.565)	(0.535)	(0.004)	(0.028)	(0.434)	(0.411)	
R4	153.8	0.046 ^c^	0.416^ab^	9.221^a^	9.683^a^	0.071	0.640	14.179	14.890	5.2
		(0.001)	(0.041)	(0.465)	(0.505)	(0.002)	(0.063)	(0.715)	(0.776)	
R5	9.7	0.121^d^	0.580^b^	6.649^c^	7.351^b^	0.012	0.056	0.647	0.716	1.8
		(0.002)	(0.044)	(0.103)	(0.135)	(0.001)	(0.004)	(0.010)	(0.013)	
R6	32.0	0.117^d^	0.511^ab^	9.012^a^	9.639^a^	0.037	0.163	2.882	3.083	7.5
		(0.010)	(0.117)	(0.614)	(0.721)	(0.003)	(0.037)	(0.196)	(0.231)	
Total	355.05	0.083	0.418	8.163	8.664	0.223	1.369	29.902	31.494	5.5
		(0.001)	(0.029)	(0.475)	(0.504)	(0.001)	(0.085)	(1.654)	(1.74)	

^†^Data represent the mean and standard deviation (parentheses); data with the same lower letter in the same column indicates no significant differences at the P = 0.05 level.

^‡^R1, temperate semi-humid meadow steppe regions; R2, temperate semi-arid steppe and desert steppe regions; R3, temperate and warm temperate arid desert and mountain steppe regions; R4, Qinghai-Tibet alpine regions; R5, warm temperate semi-humid and semi-arid shrub-tussock regions; R6, subtropical and tropical shrub-tussock regions (seeing Fig. 1).

^§^AGBC, carbon density of the above-ground biomass; BGBC, carbon density of the below-ground biomass.

**Table 2:** Estimation of carbon density and storage of Chinese grasslands using three different grassland classifications (different scales).

Grassland typegroups	Area (10^6^ha)	C density (kg C m^−2^)				C storage (Pg C)				CV
		AGBC‡	BGBC	SOC	Ecosystem	AGBC	BGBC	SOC	Ecosystem	(%)
Steppe	138.20	0.059^a†^	0.354^a^	6.497^ac^	6.910^ac^	0.081	0.489	8.979	9.549	5.2
		(0.010)†	(0.109)	(0.474)	(0.356)	(0.013)	(0.151)	(0.655)	(0.492)	
Meadow	119.19	0.067^a^	0.508^b^	11.633^b^	12.208^b^	0.080	0.605	13.866	14.551	5.5
		(0.008)	(0.041)	(0.622)	(0.668)	(0.009)	(0.049)	(0.741)	(0.797)	
Desert	61.61	0.048^a^	0.075^c^	5.617^a^	5.740^a^	0.029	0.047	3.460	3.536	7.6
		(0.008)	(0.021)	(0.460)	(0.438)	(0.005)	(0.013)	(0.283)	(0.270)	
Shrub-tussock	34.77	0.139^b^	0.718 ^d^	7.292^c^	8.148^c^	0.048	0.250	2.535	2.833	13.0
		(0.022)	(0.022)	(1.013)	(1.057)	(0.008)	(0.008)	(0.352)	(0.367)	
Total	353.77	0.078	0.414	7.760	8.251	0.239	1.390	28.841	30.470	2.2
		(0.006)	(0.041)	(0.157)	(0.182)	(0.018)	(0.204)	(0.135)	(0.283)	

^†^Data represent the mean and standard deviation (parentheses); data with the same lower letter in the same column indicate no significant differences at the P = 0.05 level.

^‡^AGBC, carbon density of the above-ground biomass; BGBC, carbon density of the below-ground biomass.

**Table 3:** Estimation of carbon density and storage from different studies of Chinese grasslands†

No.	Data and methods	Period	Area(10^6^ha)	Vegetation C density (kg C m^−2^)			Vegetation C storage(Pg C)			SOCdensity (kg C m^−2^)	SOC storage (Pg C)	Ecosystemstorage (Pg C) C	Reference
				AGBC‡	BGBC	Vegetation	AGBC	BGBC	Vegetation				
1	Global mean C density	1980s	405.9			1.15			4.66		53.72	58.38	2
2	Global mean C density	1980s	299.0			1.15			3.06	13.10	41.03	44.09	22
3	Grassland resource survey	1980s	299.0	0.06			0.13						12
4	Grassland resource survey	1980s	331.4	0.05		0.35	0.15		1.15				10
5	Grassland resource survey& NDVI	1980s	331.4	0.04	0.27	0.32	0.15	0.90	1.05				13
6	Grassland resource survey &NDVI	1980s	334.1	0.04	0.27	0.32	0.15	0.91	1.05				14
7	CEVSA &NDVI	1981–1998	167.0			0.34			0.56	9.99	16.70	17.26	15
8	Grassland resource survey& Field-measured data	1980s 2003–2004	331.0	0.12	0.88	1.00	0.39	2.92	3.32				21
9	Second soil survey	1980s	249.3							13.54	37.70		26
10	Integrating data	1980s–2000s	331.4			0.82			2.72	12.99	43.00	45.51	35
11	Field-measured data	2003–2014	355.05	0.08	0.42	0.50	0.23	1.38	1.61	7.96	29.37	30.98	This study

^†^The soil depths for the estimation of SOC density and storage are all approximately 100 cm in this table

^‡^AGBC, carbon density of the above-ground biomass; BGBC, carbon density of the below-ground biomass.

**Table 4:** Six integrative methods to estimate C storage in Chinese grasslands.

Approaches	No.method	Sub-No.	Selected methods	Assumption
Spatial interpolation	M1	1	Kriging interpolation	Grasslands vary gradually with climate and have a continuous distribution in China. Theoretically, biomass and C storage from one sampling site may be the most similar to these of the nearest site because of more similar hydrothermal conditions. On the basis of this assumption, the methods of spatial interpolation can be used to estimate the C storage of grasslands in China.
	M2	2	Inverse DistanceWeighted interpolation	
	M3	3	Empirical BayesianKriging interpolation	
Grassland classification	M4	1	5 grassland types	Grasslands show different characteristics resulting from climate (temperature and precipitation), topography, and soil. Grasslands therefore can be artificially divided into different types to depict the collective characteristics. On the basis of this assumption, C storage in Chinese grasslands can be calculated based on grassland type and corresponding area at different scales.
	M5	2	18 grassland types	
	M6	3	32 grassland subtypes	

## References

[b1] Scrulock J. M. O., Johnson K. & Olson R. Estimating net primary productivity from grassland biomass dynamics measurements. Global Change Biol. 8, 736–753 (2002).

[b2] Ni J. Carbon storage in terrestrial ecosystems of China: estimates at different spatial resolutions and their response to climate change. Climatic Change 49, 339–358 (2001).

[b3] Fang J. Y., Liu G. H. & Xu S. L. Carbon Storage in Terrestrial Ecosystem of China. In: Hot Spots in Modern Ecology (eds Wang R.S., Fang J.Y., Gao L. *et al.*) 251–277(in Chinese) (China Science and Technology Press, 1996).

[b4] He N. P., Yu Q., Wu L., Wang Y. S. & Han X. G. Carbon and nitrogen store and storage potential as affected by land-use in a *Leymus chinensis* grassland of northern China. Soil Biology & Biochemistry 40, 2952–2959 (2008).

[b5] He N. P. *et al.* Land-use impact on soil carbon and nitrogen sequestration in typical steppe ecosystems, Inner Mongolia. Journal of Geographical Sciences 22, 859–873 (2012).

[b6] Wang S. P. *et al.* Management and land use change effects on soil carbon in northern China's grasslands: a synthesis. Argiculture Ecosystems & Environment 142, 329–340 (2011).

[b7] He N. P. *et al.* Grazing intensity impacts soil carbon and nitrogen storage of continental steppe. Ecosphere 2, 1–10 (2011).

[b8] Jones M. B. & Donnelly A. Carbon sequestration in temperate grassland ecosystems and the influence of management, climate and elevated CO_2_. New Phytologist 164, 423–439 (2004).

[b9] Gower S. T. Patterns and mechanisms of the forest carbon cycle. Annual Review of Environment and Resources 28, 169–204 (2003).

[b10] Fang J. Y., Guo Z. D., Piao S. L. & Chen A. P. Terrestrial vegetation carbon sinks in China, 1981—2000. Science in China Series D: Earth Sciences 50, 1341–1350 (2007).

[b11] Houghton R. A. Aboveground forest biomass and the global carbon **b**alance. Global Change Biol. 11, 945–958 (2005).

[b12] Ni J. Forage yield-based carbon storage in grasslands of China. Climatic Change 67, 237–246 (2004).

[b13] Yang Y. H., Fang J. Y., Ma W. H., Guo D. L. & Mohammat A. Large-scale pattern of biomass partitioning across China's grasslands. Global Ecology and Biogeography 19, 268–277 (2010a).

[b14] Piao S. L., Fang J. Y., He J. S. & Xiao Y. Spatial distribution of grassland biomass in China. Acta Phytoecologica Sinica 28, 491–498 (in Chinese) (2004).

[b15] Piao S. L., Fang J. Y., Zhou L. M., Tan K. & Tao S. Changes in biomass carbon stocks in China's grasslands between 1982 and 1999. Global Biogeochem. Cy. 21, 2; 10.1029/2005GB002634 (2007).

[b16] Li K. R., Wang S. Q. & Cao M. K. Vegetation and soil carbon storage in China. Science in China Series D: Earth Sciences 47, 49–57 (2004).

[b17] Yang Y. H. *et al.* Soil carbon stock and its changes in northern China's grasslands from 1980s to 2000s. Global Change Biol. 16, 3036–3047 (2010b).

[b18] Fang J. Y., Yang Y. H., Ma W. H., Mohammat A. & Shen H. H. Ecosystem carbon stocks and their changes in China's grasslands. Science China Life Science 53, 757–765 (2010). 10.1007/s11427-010-4029-x 20697865

[b19] Sun Z. G., Sun C. M., Li J. L. & Chen Y. Z. Retrospect and prospect of carbon circle mechanism and carbon storage calculation of grassland ecosystem in China. Pratacultural Science 28, 1611–1616(in Chinese) (2011).

[b20] Hasituya. Estimation of XilinGol grassland biomass carbon stock. *Inner Mongolia: Inner Mongolia Normal University*, 15–16(in Chinese) (2012).

[b21] Fan J. W. *et al.* Carbon storage in the grasslands of China based on field measurements of above- and below-ground biomass. Climatic Change 86, 375–396 (2008).

[b22] Ni J. Carbon storage in grasslands of China. Journal of Arid Environment 50, 205–218 (2002).

[b23] Yang Y. H., Mohammat A., Feng J. M., Zhou R. & Fang J. Y. Storage, patterns and environmental controls of soil organic carbon in China. Biogeochemistry 84, 131–141 (2007).

[b24] Jobbagy E. G. & Jackson R. B. The vertical distribution of soil organic carbon and its relation to climate and vegetation. Ecological Applications 10, 423–436 (2000).

[b25] DAHV& GSAHV. Rangeland Resources of China. 147–489(in Chinese) (China Science and Technology Press,1996).

[b26] Xie Z. B. *et al.* Soil organic carbon stocks in China and changes from 1980s to 2000s. Global Change Biol. 13, 1989–2007 (2007).

[b27] Yang T. T., Wu X. H., Wang J. T., Li P. & Shi H. X. Estimation of carbon storage in grassland ecosystem in China. Journal of Arid Land Resources and Environment 26, 127–130(in Chinese) (2012).

[b28] Fang C. M. Relatively stable carbon stocks in China's grasslands. Science China Life Science 54, 490–492 (2011). 10.1007/s11427-011-4169-7 21574049

[b29] CISNR (Commission for Integrated Survey of Natural Resources, Chinese Academy of Sciences).Map of Grassland Resources in China (1:4M). (Science Press, 1996).

[b30] Wang S. Q., Zhou C. H., Li K. R., Zhu S. L. & Huang F. H. Analysis on spatial distribution characteristics of soil organic carbon reservoir in China. Acta Geographica Sinica 55, 533–544(in Chinese) (2000).

[b31] Chai H. *et al.* Vertical distribution of soil carbon, nitrogen, and phosphorus in typical Chinese terrestrial ecosystems. Chinese Geographical Sciences 25, 10.1007/s11769-015-0756-z (2015).

[b32] Zhang X. S. Vegetation Map of the People's Republic of China(1:1000 000). (in Chinese) (Geology Publishing House, 2007).

[b33] Liang E. *et al.* Terrestrial soil organic carbon storage in China: Estimates and uncertainty. Soil and Fertilizer Sciences in China 6, 75–79(in Chinese) (2010).

[b34] Oren R. *et al.* Estimating the uncertainty in annual net ecosystem carbon exchange: spatial variation in turbulent fluxes and sampling errors in eddy-covariance measurements. Global Change Biol. 12, 883–896 (2006).

[b35] Yu G.R., Li X.R., Wang Q.F. & Li S.G. Carbon storage and its spatial pattern of terrestrial ecosystem in China. J. Resour. Ecol. 1, 97–109 (2010).

