# Supplementary material for: Carbon storage in Chinese grassland ecosystems: Influence of different integrative methods
Source: Sci Rep. 2016 Feb 17;6:21378. doi: 10.1038/srep21378 (PMC4756709; doi:10.1038/srep21378)
Supplement: Supplementary Information [file srep21378-s1.doc]

**Carbon storage in Chinese grassland ecosystems: Influence of different** **integrative methods**

Anna Ma, Nianpeng He ^*^, Guirui Yu ^*^, Ding Wen, Shunlei Peng

Key Laboratory of Ecosystem Network Observation and Modeling, Institute of Geographic Sciences and Natural Resources Research, Chinese Academy of Sciences, Beijing 100101, China

* For correspondence. E-mail: [henp@igsnrr.ac.cn](mailto:henp@igsnrr.ac.cn); [yugr@igsnrr.ac.cn](mailto:yugr@igsnrr.ac.cn)

**Supplementary Fig. S1**


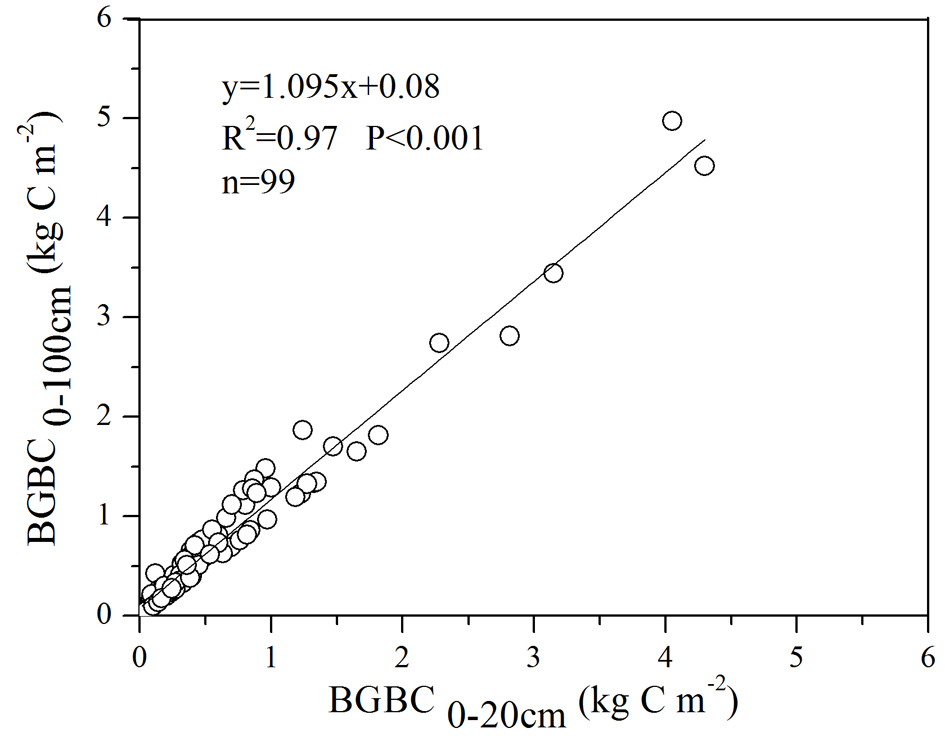


Fig. S1 Relationships between the measured values of C density of below-ground biomass

(BGBC, kg C m^-2^) in the 0–20 cm and 0–100 cm depth soil layers.

**Supplementary Fig. S2**


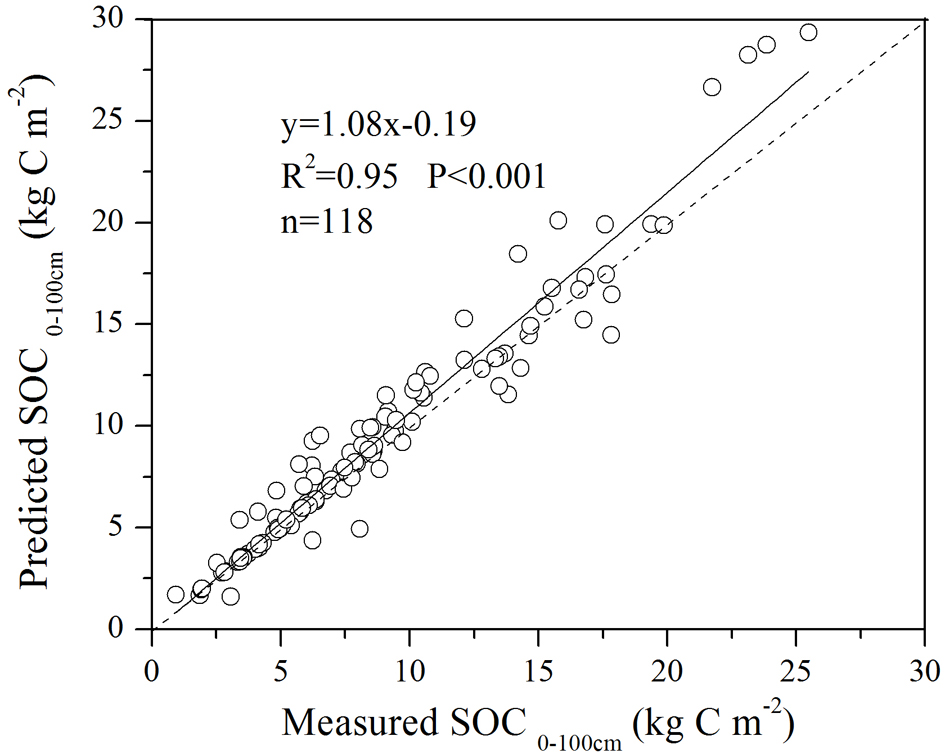


Fig. S2 Relationships between the measured and predicted values of SOC density (SOC, kg C m^-2^) in the 0–100 cm soil layer. The power function was used to fit the 0–100 cm SOC from soil surface data, where the power functions were derived from Chai et al. ^31^

**Supplementary Appx.S1**

Appx.S1 Data sources for the published and unpublished data presented in this paper

**Part 1 Published paper**

Bai, C.H. (2008) Effects of land use pattern and grazing schedule on C and N reserves in 3 grassland communities in Inner Mongolian steppe [D]. *Beijing: The Institute of Botany, CAS* (in Chinese).

Bai, Y.F., Li, L.H., Wang, Q.B., Zhang, L.X., Zhang, Y. & Chen, Z.Z. (2000) Changes in plant species diversity and productivity along gradients of precipitation and elevation in the Xilin River basin, Inner Mongolia. *Acta Phytoecologica Sinica*, **24**, 667-673(in Chinese).

Bai, Y.F., Wu, J.G., Xing, Q., Pan, Q.M., Huang, J.H., Yang, D.L. & Han, X.G. (2008) Primary production and rain use efficiency across a precipitation gradient on the Mongolia Plateau. *Ecology,* **89**, 2140-2153.

Bai, Y.F., Wu, J.G., Clark, C.M., Pan, Q.M., Zhang, L.X., Chen, S.P., Wang, Q.B., Han, X. & Wisley, B. (2012) Grazing alters ecosystem functioning and C:N:P stoichiometry of grasslands along a regional precipitation gradient. *Journal of Applied Ecology*, **49**, 1204-1215.

Bao, L.D., Xu, W.F., Wang, Z.W., Wang, J. & Han, G.D. (2012) Carbon sequestration of *Stipa breviflora* desert steppe under different grazing intensities. *Journal of Inner Mongolia Agricultural University,* **33**, 94-99 (in Chinese).

Cai, X.B., Yu, B.Z., Peng, Y.L. & Liu, H.M. (2013) The changes of soil organic carbon and carbon management index in alpine steppe. *Acta Ecologica Sinica*, **33**, 7748-7755 (in Chinese).

Cai, X.B., Peng, Y.L., Wei, S.Z. & Yu, B.Z. (2014) Variation of organic carbon and humus carbon in alpine steppe soil and functions of microorganisms therein. *Acta Pedologica Sinica,* **51**, 166-176(in Chinese).

Cao, G.M., Tang, Y.H., Ma, W.H., Wang, Y.S., Li, Y.N. & Zhao, X.Q. (2004) Grazing intensity alters soil respiration in an alpine meadow on the Tibetan plateau. *Soil Biology and Biochemistry,* **36**, 237-243.

Cao, G.M., Long, R.J., Zhang, F.W., Li, Y.K., Lin, L., Guo, X.W., Han, D.R. & Li, J. (2010) A method to estimate carbon storage potential in alpine *Kobresia* meadows on the Qinghai-Tibetan Plateau. *Acta Ecologica Sinica,* **30**, 6591-6597 (in Chinese).

Cao, J.J., Yeh, E.T., Holden, N.M., Yang, Y.Y. & Du, G.Z. (2013) The effects of enclosures and land-use contracts on rangeland degradation on the Qinghai–Tibetan plateau. *Journal of Arid Environments*, **97**, 3-8.

Cao, S.K., Chen, K.L., Cao, G.C., Zhu, J.F., Lu, B.L. & Wang, J.M. (2014) Influence of grassland gegradation on the soil carbon density of the *Kobresia pygmaes* meadow in the Qinghai Lake Basin. *Research of Soil and Water Conservation*, **21**,71-75 (in Chinese).

Cao, S.K., Chen, K.L., Cao, G.C., Zhu, J.F., Lu, B.L., Zhang, T. & Wang, J.M. (2014) Characteristics of soil carbon density distribution of the *Kobresia humilis* meadow in the Qinghai Lake basin. *Acta Ecologica Sinica,* **34**, 482-490(in Chinese).

Chang, X.F. (2012) Soil organic carbon in the alpine grasslands on the Three-River source region: spatial variations and controlling factors [D]. *Qinghai: Northwest Institute of Plateau Biology, CAS* (in Chinese).

Chen, F.R. (2012) Research on the influences of disturbances types on vegetation and soil in the typical steppe of Loess region [D]. *Shanxi: Institute of soil and water conservation, CAS and MWR* (in Chinese).

Chen, F.R., Cheng, J.M., Liu, W., Zhu, R.B., Yang, X.M., Zhao, X.Y. & Su, J.S. (2013) Effects of different disturbances on diversity and biomass of communities in the typical steppe of loess region. *Acta Ecologica Sinica,* **33**, 2856-2866 (in Chinese).

Chen, J., Cao, J.J., Wei, Y.L., Zhang, B.C., Zhu, B.W. & Ma, Z.T. (2014) Primary study on the allocation pattern of grassland biomass under soil water gradient of bird island in Qinghai lake. *Agricultural Research in the Arid Areas*, **32**, 202-208 (in Chinese).

Chen, M.D., Huang, X.D., Hou, X.M., Feng, Q.S., Yu, H., Guo, Z.G. & Liang, T.G. (2013) Dynamic monitoring of biomass and vegetation coverage in rodent damaged grassland regions of Qinghai Province, China. *Acta Pratacurae Sinica*, **22**, 247-256 (in Chinese).

Chen, Q.S. (2002) Study on the spatio-temporal variation of soil respiration and the controlling factors in the Xilin River basin of Inner Mongolia[D]. *Beijing: The Institute of Botany, Chinese Academy of Sciences* (in Chinese).

Chen, X.B., Zheng, H., Zhang, W., He, X.Y., Li, L., Wu, J.S., Huang, D.Y. & Su, Y.R. (2014) Effect of land cover on soil organic carbon stock in a Karst landscape with discontinuous soil distribution. *Journal of Mountain Science,* **11**, 774-781.

Chen, Y.P., Li, Y.Q., Awada, T., Han, J.J. & Luo, Y.Q. (2012) Carbon sequestration in the total and light fraction soil organic matter along a chronosequence in grazing exclosures in a semiarid degraded sandy site in China. *Journal of Arid Land*, **4**, 411-419.

Chen, Y.P., Li, Y.Q., Zhao, X.Y., Awada, T., Shang, W. & Han, J.J. (2012) Effects of grazing exclusion on soil properties and on ecosystem carbon and nitrogen storage in a sandy rangeland of Inner Mongolia, northern China. *Environment Manage,* **50**, 622-32.

Chen, Y.Y., Yang, K., Tang, W.J., Qin, J. & Zhao, L. (2012) Parameterizing soil organic carbon’s impacts on soil porosity and thermal parameters for Eastern Tibet grasslands. *Science China Earth Sciences,* **55**, 1001-1011.

Cheng, J., Wu, G.L., Zhao, L.P., Li, Y., Li, W. & Cheng, J.M. (2011) Cumulative effects of 20-year exclusion of livestock grazing on above-and belowground biomass of typical steppe communities in arid areas of the Loess Plateau, China. *Plant Soil Environment,* **57**, 40-44.

Cheng, J.M., Cheng, J. & Yang, X.M. (2011) Grassland vegetation and soil carbon sequestration in the Loess Plateau. *Journal of Natural Resources*, **26**, 401-411 (in Chinese).

Cheng, J.M., Jing, Z.B., Jin, J.W. & Gao, Y. (2014) Restoration and utilization mechanism of degraded grassland in the semi-arid region of Loess Plateau. *Scientia Sinica Vitae,* **44**, 267-279 (in Chinese).

Cheng, X.L., An, S.Q., Liu, S.R. & Li, G.Q. (2004) Micro-scale spatial heterogeneity and the loss of carbon, nitrogen and phosphorus in degraded grassland in Ordos Plateau, northwestern China. *Plant and Soil,* **259**, 29-37.

Cui, X.Y., Wang, Y.F., Niu, H.S., Wu, J., Wang, S.P., Schnug, E., Rogasik, J., Fleckenstein, J. & Tang, Y.H. (2005) Effect of long-term grazing on soil organic carbon content in semiarid steppes in Inner Mongolia. *Ecological Research*, **20**, 519-527.

Deng, L., Sweeney, S. & Shangguan, Z.P. (2014) Grassland responses to grazing disturbance: plant diversity changes with grazing intensity in a desert steppe. *Grass and Forage Science,* **69**, 524-533.

Deng, L., Zhang, Z.N. & Shangguan, Z.P. (2014) Long-term fencing effects on plant diversity and soil properties in China. *Soil and Tillage Research,* **137**, 7-15.

Deng, L., Wang, K.B., Li, J.P., Zhou Ping Shangguan & Sweeney, S. (2014) Carbon storage dynamics in Alfalfa (Medicago sativa) fields in the Hilly-Gully region of the Loess Plateau, China. *CLEAN - Soil, Air, Water,* **42**, 1253-1262.

Ding, X.H., Gong, L., Wang, D.B., Wu, X. & Liu, G.H. (2012) Grazing effects on eco-stoichiometry of plant and soil in Hulunbeir, Inner Mogolia. *Acta Ecologica Sinica,* **32**, 4722-4730 (in Chinese).

Ding, X.H., Luo, S.Z., Liu, J.W., Li, K. & Liu, G.H. (2012) Longitude gradient changes on plant community and soil stoichiometry characteristics of grassland in Hulunbeir. *Acta Ecologica Sinica,* **32**, 3467-3476(in Chinese).

Dong, Q.M., Zhao, X.Q., Ma, Y.S., Shi, J.J., Wang, Y.L., Li, S.X., Yang, S.H., Wang, L.Y. & Sheng, L. (2012) Influence of grazing on biomass, growth ratio and compensatory effect of different plant groups in *Kobresia parva* meadow. *Acta Ecologica Sinica,* **32**, 2640-2650(in Chinese).

Dong, S.K., Wen, L., Li, Y.Y., Wang, X.X., Zhu, L. & Li, X.Y. (2012) Soil-quality effects of grassland degradation and restoration on the Qinghai-Tibetan Plateau. *Soil Science Society of America Journal,* **76**, 2256-2264.

Dong, X.Y., Fu, H., Li, X.D., Niu, D.C., Guo, D. & Li, X.D. (2010) Effects on plant biomass and CNP contents of plants in grazed and fenced steppe grassland of the Loess Plateau. *Acta Prataculturae Sinica,* **19**, 175-182 (in Chinese).

Du, F., Liang, Z.S., Xu, X.X., Shan, L. & Zhang, X.C. (2007) The community biomass of abandoned farmland and its effects on soil nutrition in the Loess Hilly Region of Northern Shaanxi,China. *Acta Ecologica Sinica,* **27**, 1673-1683 (in Chinese).

Fan, Y.G. (2008) Study on organic carbon storage and the relation with environment factors in Bayanbulak [D]. *Xinjiang: Xinjiang Institute of Ecology and Geography, CAS* (in Chinese).

Fan, Y.G., Hu, Y.K., Li, K.H., Yu, J.H. & Wang, X. (2008) Effect of different disturbances on the diversity and biomass of the Phytobiocoenoses in alpine steppes. *Arid Zone Research,* **25**, 532-536 (in Chinese).

Fan, Y.J., Hou, X.Y., Shi, H.X. & Shi, S.L. (2013) Effects of grazing and fencing on carbon and nitrogen reserves in plants and soils of alpine meadow in the three headwater resource regions. *Russian Journal of Ecology,* **44**, 80-88.

Fan, Y.M., Wu, H.Q., Jin, G.L., Liu, W. & Adelieti (2009) Change of plant community character and soil nutrients under different utilization models of spring-autumn steppe. *Xinjiang Agricultural Sciences,* **5**, 985-990(in Chinese).

Fang, K., Song, N.P., Wei, L. & An, H. (2012) The effect of different grazing systems on aboveground biomass and interspecific relationships in desert steppe. *Acta Prataculturae Sinica,* **21**, 12-22 (in Chinese).

Ganjurjav, Guo, Y.Q., Gao, Q.Z., Duan, M.J., Wan, Y.F., Li, Y.E. & Luobu, D. (2013) A study on optimal grazing rates in *Stipapurpurea* alpine grassland in Northern Tibet. *Acta Prataculturae Sinica,* **22**, 130-137 (in Chinese).

Gao, C. (2007) Study on the characteristics of soil organic matter of alpine meadow under different degradation degrees in eastern Qilian Mountains and its effect on productivity [D]. *Gansu: Gansu Agricultural University* (in Chinese).

Gao, H.N., Zhang, Y., Qin, J.H. & Wang, Z.J. (2014) Organic carbon distribution and enzyme activities of different degraded meadows soil in upstream of Heihe of Qinlian mountains. *Acta Agrestia Sinica,* **22**, 283-290 (in Chinese).

Gao, L. (2013) Soil organic carbon storage and the influencing factors in the typical grassland ecosystem in China [D]. *Beijing: China University of Geosciences* (in Chinese).

Gao, N.N., Chen, J., Zhang, P.L., Liu, S.J., Xu, Y.F. & Hu, T.M. (2014) Effects of grazing intensity on the spatial distribution of aboveground biomass of alpine *Kobresia* meadow in Tibetan. *Acta Agrestia Sinica,* **22**, 255-260 (in Chinese).

Gao, Y. & Cheng, J. (2013) Spatial and temporal variations of grassland soil organic carbon and total nitrogen following grazing exclusion in semiarid Loess Plateau, Northwest China. *Acta Agriculturae Scandinavica, Section B - Soil & Plant Science,* **63**, 704-711.

Gao, Y.H., Luo, P., Wu, N., Chen, H. & Wang, G.X. (2007) Grazing intensity impacts on carbon sequestration in an alpine meadow on the eastern Tibetan Plateau. *Journal of Agriculture and BIological Sciences,* **3**, 642-647.

Gao, Y.Z., Giese, M., Lin, S., Sattelmacher, B., Zhao, Y. & Brueck, H. (2008) Belowground net primary productivity and biomass allocation of a grassland in Inner Mongolia is affected by grazing intensity. *Plant and Soil,* **307**, 41-50.

Geng, Y., Wang, Y.H., Yang, K., Wang, S.P., Zeng, H., Baumann, F., Kuehn, P., Scholten, T. & He, J.S. (2012) Soil respiration in Tibetan alpine grasslands: belowground biomass and soil moisture, but not soil temperature, best explain the large-scale patterns. *PLoS One,* **7**, 1-12.

Gong, X., Brueck, H., Giese, K.M., Zhang, L., Sattelmacher, B. & Lin, S. (2008) Slope aspect has effects on productivity and species composition of hilly grassland in the Xilin River Basin, Inner Mongolia, China. *Journal of Arid Environments*, **72**, 483-493.

Gu, W.R., Zhang, X.H., Zhu, J.Z., Sun, Z.J., Mu, X.Y. & Wang, X.J. (2013) Impact of seasonal rest grazing on plant community quantity characteristics under different grazing intensities. *Xinjiang Agricultural Sciences,* **50**, 1145-1149 (in Chinese).

Guo, Y.J., Han, L., Li, G.D., Han, J.D., Wang, G.L., Li, Z.Y. & Wilson, B. (2012) The effects of defoliation on plant community, root biomass and nutrient allocation and soil chemical properties on semi-arid steppes in northern China. *Journal of Arid Environments,* **78**, 128-134.

Ha, Q. (2012) The carbon storage of different grassland patterns in Saihanwula national nature reserve [D]. *Inner: Inner Mongolia Agricultural University* (in Chinese).

Han, B., Fan, J.W. & Zhong, H.P. (2006) Grassland biomass of communities along gradients of the Inner Mongolia grassland transect. *Journal of Plant Ecology,* **30**, 553-562 (in Chinese).

Han, D.R. (2012) The carbon storage of alpine grassland in Qinghai-Tibetan plateau and its relationship to the climatic factors[D]. *Qinghai: Northwest institute of plateau biology, CAS* (in Chinese).

Han, G.D., Hao, X.Y., Zhao, M.L., Wang, M.J., Ellert, B.H., Willms, W. & Wang, M.J. (2008) Effect of grazing intensity on carbon and nitrogen in soil and vegetation in a meadow steppe in Inner Mongolia. *Agriculture, Ecosystems & Environment*, **125**, 21-32.

Han, J.J., Li, Y.Q., Wang, S.K., Luo, Y.Q. & Lian, J. (2014) Characteristics of soil organic carbon and total nitrogen under different land use types in Naiman banner. *Journal of Arid Land Resources and Environment,* **28**, 37-42 (in Chinese).

Han, L., Guo, Y.J., Han, J.G., Guo, Y.J. & Tang, H. (2010) A study on the diversity and aboveground biomass in aLeymus chinensis meadow steppe community under different cutting intensities. *Acta Prataculture Sinica*, **19**, 70-75 (in Chinese).

Han, W.J., Hou, X.Y., Olokhnuud, C.L. & Michael, P.S. (2014) The characteristics ofplant communities along East Eurasian steppe transect. *Journal of Integrative Agriculture,* **13**, 1157-1164.

Han, X.W., Tsunekawa, A., Tsubo, M. & Li, S.Q. (2011) Aboveground biomass response to increasing nitrogen deposition on grassland on the northern Loess Plateau of China. *Acta Agriculturae Scandinavica, Section B - Plant Soil Science,* **61**, 112-121.

He, H.Y., Su, J.Q., Huang, L., Jia, R.L. & Li, X.R. (2011) Effects of fire on the structure of herbage synusia vegetation in desertified steppe, North China. *Acta Ecologica Sinica,* **11**, 364-370 (in Chinese).

He, N.P., Yu, Q., Wu, L., Wang, Y.S. & Han, X.G. (2008) Carbon and nitrogen store and storage potential as affected by land-use in a Leymus chinensis grassland of northern China. *Soil Biology and Biochemistry,* **40**, 2952-2959.

He, N.P., Zhang, Y.H., Yu, Q., Chen, Q.S., Pan, Q.M., Zhang, G.M. & Han, X.G. (2011) Grazing intensity impacts soil carbon and nitrogen storage of continental steppe. *Ecosphere,* **2**, 1-10.

Hu, C.J., Liu, G.H., Fu, B.J., Chen, L.D., Liu, Y.H. & Guo, L. (2014) Soil carbon stock and flux in plantation forest and grassland ecosystems in Loess Plateau, China. *Chinese Geographical Science,* **24**, 423-435.

Hu, E.C. (2009) Study on the dynamics and relationship between the standing live aboveground biomass and stocking rate in different scales in the steppe grassland[D]. *Inner Mongolia: Inner Mongolia University* (in Chinese).

Huang, D.Q., Yu, L., Zhang, Y.S. & Zhao, X.Q. (2011) Belowground biomass and its relationship to environmental factors of natural grassland on the northern slopes of the Qilian Mountains. *Acta Prataculturae Sinica,* **20**, 1-10 (in Chinese).

Huang, R., Wang, H., Wang, H. & Shi, X.W. (2014) Effects of enclosure year on soil physicochemical properties of sandy grassland. *Journal of Soil and Water Conservation,* **28**, 183-197 (in Chinese).

Ji, S.J., Geng, Y., Li, D.F. & Wang, G.H. (2009) Plant coverage is more important than species richness in enhancing aboveground biomass in a premature grassland, northern China. *Agriculture, Ecosystems & Environment,* **129**, 491-496.

Jin, Y.X., Xu, B., Yang, X.C., Li, J.Y., Ma, H.L., Gao, T. & Yu, H.D. (2013) Below-ground biomass and features of environmental factors in the degree of grassland dertification. *Acta Prataculture Sinica*, **22**, 44-51 (in Chinese).

Jing, Z.B., Cheng, J.M., Su, J.S., Bai, Y. & Jin, J.W. (2014) Changes in plant community composition and soil properties under 3-decade grazing exclusion in semiarid grassland. *Ecological Engineering*, **64**, 171-178.

Li, D.M., Jiao, F., Lei, B. & Zhang, Z. (2014) Aboveground biomass production and soil moisture characteristics of different herb communities in the Loess Hilly-gully region. *Science of Soil and Water Conservation,* **12**, 33-37 (in Chinese).

Li, F., Yu, P.J., Shen, X.J., Song, Y.T., Li, Q., Zhang, H.Y. & Zhou, D.W. (2014) Community productivity and soil carbon sequestration after *Melilotus of ficinalis* and *Medicago falcata* reseeding on degraded grassland. *Pratacultural Science,* **31**, 361-366 (in Chinese).

Li, F.R., Zhao, W.Z., Liu, J.L. & Huang, Z.G. (2008) Degraded vegetation and wind erosion influence soil carbon, nitrogen and phosphorus accumulation in sandy grasslands. *Plant and Soil,* **317**, 79-92.

Li, G., Jiang, G.M., Li, Y.G. & Liu, M.Z. (2011) Biomass carbon storage and net primary production in different habitats of Hunshandake Sandland, China. *Acta Ecologica Sinica,* **31**, 217-224.

Li, K.H., Wang, W.L., Hu, Y.K., Gao, G.G., Gong, Y.M. & Yi, W. (2008) Relationships between belowground biomass of alpine grassland and environmental factors along an altitude gradient. *Chinese Journal of Applied Ecology*, **19**, 2364-2368 (in Chinese).

Li, L., Yao, Y.F., Qin, F.C. & Guo, Y.F. (2014) Spatial variations of organic carbon of Huanghuadianzi watershed in Chifeng. *Acta Scientiae Circumstantiae,* **34**, 742-748 (in Chinese).

Li, N., Wang, G.X., Yang, Y., Gao, Y.H., Liu, L.A. & Liu, G.S. (2011) Short-term effects of temperature enhancement on community structure and biomass of alpine meadow in the Qinghai-Tibet Plateau. *Acta Ecologica Sinica,* **31**, 895-905 (in Chinese).

Li, Q., Zhou, D.W., Jin, Y.H., Wang, M.L., Song, Y.T. & Li, G.D. (2013) Effects of fencing on vegetation and soil restoration in a degraded alkaline grassland in northeast China. *Journal of Arid Land,*, **6**, 478-487.

Li, Q., Yang, J., Song, B.Y., Ma, W.H., Zhao, L.Q., Zhang, L.X. & Hou, H. (2014) The impacts of different enclosure durations on degraded Stipa grandis grassland productivity and soil carbon and nitrogen storage. *Chinese Journal of Ecology,* **33**, 896-901 (in Chinese).

Li, T. (2013) Research on soil and vegetation carbon storage of grassland under different land use and degree in Aba Pastoral Areas. *Sichuan: Sichuan Agricultural University* (in Chinese).

Li, W., Huang, H.Z., Zhang, Z.N. & Wu, G.L. (2011) Effects of grazing on the soil properties and C and N storage in relation to biomass allocation in an alpine meadow. *Journal of Soil Science and Plant Nutrition,* **11**, 27-39.

Li, W.H. (2005) Grassland ecosystem production and leaf area index in the Gonghe Basin, Qinghai province [D]. *Beijing: Institute of Geographic Sciences and Natural Resources Research, CAS* (in Chinese).

Li, W.J., Wang, Z., Han, Q.F., Ren, C.H., Yan, M.K., Zhang, P., Jia, Z.K. & Yang, B.P. (2013) Evaluation on carbon sequestration effects of artificial alfalfa pastures in the Loess Plateau area. *Acta Ecologica Sinica*, **33**, 7467-7477 (in Chinese).

Li, X.J., Zhang, X.Z., Wu, J.S., Shen, Z.X., Zhang, Y.J., Xu, X.L., Fan, Y.Z., Zhao, Y.P. & Yan, W. (2011) Root biomass distribution in alpine ecosystems of the northern Tibetan Plateau. *Environmental Earth Sciences,* **64**, 1911-1919.

Li, Y.H., Luo, T.X. & Lu, Q. (2008) Plant height as a simple predictor of the root to shoot ratio: Evidence from alpine grasslands on the Tibetan Plateau. *Journal of Vegetation Science,* **19**, 245-252.

Li, Y.J. (2013) Response of alpine grassland to land use pattern changes on Qinghai-Tibet Plateau [D]. *Gansu: Gansu Agricultural University* (in Chinese).

Li, Y.J. (2013) Effects of rest grazing on plant diversity and organic carbon storage on Stipa Baicalensis steppe in Inner Mongolia [D]. *Shenyang: Shenyang Agricultural University* (in Chinese).

Li, Y.J., Zhu, Y., Zhao, J.N., Li, G., Wang, H., Lai, X. & Yang, D.L. (2014) Effects of rest grazing on organic carbon storage in *Stipa grandis* steppe in Inner Mongolia, China. *Journal of Integrative Agriculture,* **13**, 624-634.

Li, Y.Q., Zhao, H.L., Zhao, X.Y., Zhang, T.H. & Chen, Y.P. (2006) Biomass energy, carbon and nitrogen stores in different habitats along a desertification gradient in the semiarid Horqin sandy land. *Arid Land Research and Management,* **20**, 43-60.

Li, Y.Q., Zhou, X.H., Brandle, J.R., Zhang, T.H., Chen, Y.P. & Han, J.J. (2012) Temporal progress in improving carbon and nitrogen storage by grazing exclosure practice in a degraded land area of China's Horqin Sandy Grassland. *Agriculture, Ecosystems & Environment,* **159**, 55-61.

Li, Y.Y., Shao, M.A., Zheng, J.Y. & Li, Q.F. (2007) Impact of grassland recovery and reconstruction on soil organic carbon in the northern Loess Plateau. *Acta Ecologica Sinica,* **27**, 2279-2287 (in Chinese).

Li, Y.Y., Dong, S.K., Wen, L., Wang, X.X. & Wu, Y. (2013) The effects of fencing on carbon stocks in the degraded alpine grasslands of the Qinghai-Tibetan Plateau. *Journal of Environmental Manage*, **128**, 393-399.

Li, Y.Y., Dong, S.K., Wen, L., Wang, X.X. & Wu, Y. (2014) Soil carbon and nitrogen pools and their relationship to plant and soil dynamics of degraded and artificially restored grasslands of the Qinghai–Tibetan Plateau. *Geoderma,* **213**, 178-184.

Li, Y.Y., Dong, S.K., Zhu, L., Wen, L., Li, X.Y. & Wang, X.X. (2013) Adaptation strategies of reproduction of plant community in response to grassland degradation and artificial restoration. *Acta Ecologica Sinica*, **33**, 1-9 (in Chinese).

Li, Y.Z., Fan, J.W., Zhang, L.X., Zhai, J., Liu, G.F. & Li, J. (2013) The impact of different land use and management on community composition, species diversity and productivity in a typical temperate grassland. *Acta Prataculturae Sinica*, **22**, 1-9 (in Chinese).

Lin, Y., Hong, M., Han, G.D., Zhao, M.L., Bai, Y.F. & Chang, S.X. (2010) Grazing intensity affected spatial patterns of vegetation and soil fertility in a desert steppe. *Agriculture, Ecosystems & Environment,* **138**, 282-292.

Liu, H.L., Wang, J.W., Lv, J.Y. & Wang, K. (2010) Response of grasslands conversion to croplands on soil organic carbon in Bashang area of Northern China. *African Journal of Biotechnology,* **9**, 1783-1788.

Liu, J.D. (2010) Model and romote sensing herbage yield model-To take Evenk Autonomous banner Inner Monglia as example [D]. *Inner: Inner Mongolia Agricultural University* (in Chinese).

Liu, J.L. (2013) Study on impact of mowing and grazing on vegetation and soil of typical steppe in Xilingol, Inner Mongolia[D]. *Beijing: Chinese Academy of Agricultural Sciences* (in Chinese).

Liu, L.L. (2006) A study on plant diversity and biomass of managed meadows in the Tibetan region, NW Yunnan, China [D]. *Yunnan: Xishuangbanna Tropical Botanical Garden, CAS* (in Chinese).

Liu, M., Liu, G.H., Wu, X., Wang, H. & Chen, L. (2014) Vegetation traits and soil properties in response to utilization patterns of grassland in Hulun Buir City, Inner Mongolia, China. *Chinese Geographical Science,* **24**, 471-478.

Liu, S. (2008) Study on carbon stock changes of grassland and influential factors in Da'an city[D]. *Jilin: Jilin University* (in Chinese).

Liu, W. (2011) The research of soil organic carbon storage in natural grassland ecosystem in the Loess Plateau [D]. *Shanxi: Institute of soil and water conservation, CAS and MWR* (in Chinese).

Liu, Y., Ma, Y.S., Li, S.X., Zheng, W. & Yang, S.H. (2014) Species diversity and biomass characteristics of different grain-for-green grassland in the Northern region of Qinghai lake. *Acta Agriculturae Boreali-occidentalis Sinica,* **23**, 48-52 (in Chinese).

Lu, J.F., Dong, Z.B., Li, W.J. & Hu, G.Y. (2013) The effect of desertification on carbon and nitrogen status in the northeastern margin of the Qinghai-Tibetan Plateau. *Environmental Earth Sciences,* **71**, 807-815.

Lu, X., Yan, Y., Fan, J., Cao, Y. & Wang, X. (2011) Dynamics of above- and below-ground biomass and C, N, P accumulation in the alpine steppe of Northern Tibet. *Journal of Mountain Science*, **8**, 838-844.

Luo, T.X., Brown, S., Pan, Y.D., Shi, P.L., Ouyang, H., Yu, Z.L. & Zhu, H.Z. (2005) Root biomass along subtropical to alpine gradients: global implication from Tibetan transect studies. *Forest Ecology and Management,* **206**, 349-363.

Lv, Y.H., Ma, Z.M., Zhao, Z.J., Sun, F.X. & Fu, B.J. (2014) Effects of land use change on soil carbon storage and water consumption in an oasis-desert ecotone. *Environmental Manage,* **53**, 1066-1076.

Ma, H.B., Shen, Q.J., Xie, Y.Z. & Shen, Y. (2013) Effects of the enclosing on the underground carbon storage of typical steppe in Ningxia. *Journal of Agricultural Sciences,* **34**, 1-4 (in Chinese).

Ma, T., Wu, G.L., He, Y.L., Wen, S.J., He, J.L., Liu, J.X. & Du, G.Z. (2007) The effect of simulated mowing of the fertilizing level on community production and compensatory responses on the Qinghai-Tibetan. *Acta Ecologica Sinica,* **27**, 2288-2293 (in Chinese).

Mi, J., Li, J.J., Chen, D.M., Xie, Y.C. & Bai, Y.F. (2014) Predominant control of moisture on soil organic carbon mineralization across a broad range of arid and semiarid ecosystems on the Mongolia plateau. *Landscape Ecology,* doi:10.1007/s10980-014-0040-0.

Miao, Z.H. (2013) Changes and Factors of Soil Carbon Storage in the Sanjiang Plain, Northeast China from 1980s to 2010 [D]. *Shenyang: Northeast Institute of Geography and Agroecology, CAS* (in Chinese).

Ning, F. (2009) Study on influence of disturbance manners to typical steppe vegetation and soil[D]. *Beijing: Chinese Academy of Agricultural Sciences*(in Chinese).

Niu, D., Hall, S.J., Fu, H., Kang, J., Qin, Y. & Elser, J.J. (2011) Grazing exclusion alters ecosystem carbon pools in Alxa desert steppe. *New Zealand Journal of Agricultural Research,* **54**, 127-142.

Pan, C.C., Zhao, H.L., Zhao, X.Y., Han, H.B., Wang, Y. & Li, J. (2013) Biophysical properties as determinants for soil organic carbon and total nitrogen in grassland salinization. *PLoS One*, **8**, 1-6.

Peng, H.Y., Li, X.Y. & Tong, S.Y. (2013) Effects of shrub encroachment on biomass and biodiversity in the typical steppe of Inner Mongolia. *Acta Ecologica Sinica*, **33**, 7221-7229 (in Chinese).

Qi, Y., Huang, Y.M., Wang, Y., Zhao, J. & Zhang, J.H. (2011) Biomass and its allocation of four grassland species under different nitrogen levels. *Acta Ecologica Sinica*, **31**, 5121-5129 (in Chinese).

Qi, Y.C., Dong, Y.S., Jin, Z., Peng, Q., Xiao, S.S. & He, Y.T. (2010) Spatial Heterogeneity of Soil Nutrients and Respiration in the Desertified Grasslands of Inner Mongolia, China. *Pedosphere,* **20**, 655-665.

Qi, Y.C., Peng, Q., Dong, Y.S., Xiao, S.S., Sun, L.J., Liu, X.C., He, Y.T., Jia, J.Q. & Cao, C.C. (2014) Responses of soil total organic carbon and dissolved organic carbon to simulated nitrogen deposition in temperate typical steppe in Inner Mongolia, China. *Environmental Science,* **35**, 3073-3081 (in Chinese).

Qin, Y. (2010) Effect of different land utilization on soil nutrients and grassland vegetation in alpine meadow [D]. *Gansu: Lan zhou Univeristy* (in Chinese).

Qiu, L.P., Wei, X.R., Zhang, X.C. & Cheng, J.M. (2013) Ecosystem carbon and nitrogen accumulation after grazing exclusion in semiarid grassland. *PLoS One,* **8**, 1-7.

Qu, H., Zhao, X.Y., Wang, S.K., Huang, W.D. & Mao, W. (2014) Effects of different vegetation communities on soil carbon and nitrogen contents in Urad desert steppe. *Pratacultural Science*, **31**, 355-360 (in Chinese).

Ren, A.C. (2008) Grassland biomass on North-western Plateau of Sichuan and vegetation indexes relation using landsat TM image[D]. *Yaan: Sichuan Agricultural University* (in Chinese).

Ren, J.J., Li, J., Wang, X.C. & Fang, X.Y. (2011) Soil water and nutrient characteristics of alfalfa grasslands at semi-arid and semi-arid prone to drought areas in southern Ningxia. *Acta Ecologica Sinica,* **31**, 3638-3649 (in Chinese).

Rong, Y.P., Yuan, F. & Ma, L. (2014) Effectiveness of exclosures for restoring soils and vegetation degraded by overgrazing in the Junggar Basin, China. *Grassland Science,* **60**, 118-124.

Sa, R.L. (2013) Effects of different utilization and grazing intensity on carbon storage of vegetation-soil system in typical steppe [D]. *Beijing: Chinese Academy of Agricultural Sciences* (in Chinese).

Sa, R.L., Hou, X.Y., Li, J.X., Ding, Y., Wu, X.H. & Yun, X.J. (2013) Organic carbon storage in vegetation-soil systems of typical grazing degraded steppes. *Acta Prataculturae Sinica,* **22**,18-26 (in Chinese).

Sa, W., An, L. & Sa, W. (2012) Changes in plant community diversity and aboveground biomass along with altitude within an alpine meadow on the Three-River source region. *Chinese Science Bulletin,* **57**, 3573-3577.

Shang, Z.H., Cao, J.J., Guo, R.Y., Long, R.J. & Deng, B. (2014) The response of soil organic carbon and nitrogen 10years after returning cultivated alpine steppe to grassland by abandonment or reseeding. *Catena*, **119**, 28-35.

Shang, Z.H., Cao, J.J., Guo, R.Y., Henkin, Z., Ding, L.M., Long, R.J. & Deng, B. (2014) Effect of enclosure on soil carbon, nitrogen and phosphorus of alpine desert rangeland. *Land Degradation & Development*, 10.1002/ldr.2283.

Shen, M.G., Tang, Y.H., Klein, J., Zhang, P.C., Gu, S., Shimono, A. & Chen, J. (2008) Estimation of aboveground biomass using in situ hyperspectral measurements in five major grassland ecosystems on the Tibetan Plateau. *Journal of Plant Ecology,* **1**, 247-257.

Shi, F.S., Chen, H., Wu, Y. & Wu, N. (2010) Effects of livestock exclusion on vegetation and soil properties under two topographic habitats in an alpine meadow on the eastern Qinghai-Tibetan Plateau. *Polish Journal of Ecology,* **58**, 125-133.

Shi, S.F., Wu, N. & Luo, P. (2008) Effect of temperature enhancement on community structure and biomass of subalpine meadow in Northwestern Sichuan. *Acta Ecologica Sinica,* **28**, 5286-5293 (in Chinese).

Song, L.L., Fan, J.W., Zhong, H.P. & Wang, N. (2010) Changes of biomass and species richness of grassland community along an altitude gradient in Hongchiba, Chongqing. *Acta Agrestia Sinica,* **18**, 160-166 (in Chinese).

Su, Y.Z., Zhao, H.L., Zhang, T.H. & Zhao, X.Y. (2004) Soil properties following cultivation and non-grazing of a semi-arid sandy grassland in northern China. *Soil and Tillage Research,* **75**, 27-36.

Su, Y.Z., Li, Y.L., Cui, J.Y. & Zhao, W.Z. (2005) Influences of continuous grazing and livestock exclusion on soil properties in a degraded sandy grassland, Inner Mongolia, northern China. *Catena,* **59**, 267-278.

Sun, D.S., Wesche, K., Chen, D.D., Zhang, S.H., Wu, G.L., Du, G.Z. & Comerford, N.B. (2011) Grazing depresses soil carbon storage through changing plant biomass and composition in a Tibetan alpine meadow. *Plant Soil Environment,* **57**, 271-278.

Sun, S.X., Cui, Z.M., Chen, L.B., Jia, L.J. & Wei, Z.J. (2014) Effects of seasonal regulation of grazing intensity on N and C of the main plant species and soil in desert grassland. *Chinese Journal of Grassland*, **36**, 49-54 (in Chinese).

Tang, L., Dang, X., Liu, G., Shao, C. & Xue, S. (2014) Response of artificial grassland carbon stock to management in mountain region of Southern Ningxia, China. *Chinese Geographical Science,* **24**, 436-443.

Wang, C.T. (2006) Relationship between productivity and species diversity of plant in alpine meadow [D]. *Qinghai: Northwest Plateau Institute of Biology, CAS*, 1-129 (in Chinese).

Wang, C.T., Long, R.J., Wang, Q.L., Jing, Z.C. & Shi, J.J. (2009) Changes in plant diversity, biomass and soil C, in alpine meadows at different degradation stages in the headwater region of three rivers, China. *Land Degradation & Development*, **20**, 187-198.

Wang, C.T., Cao, G.M., Wang, Q.L., Jing, Z.C., Ding, L.M. & Long, R.J. (2008) Changes in plant biomass and species composition of alpine Kobresia meadows along altitudinal gradient on the Qinghai-Tibetan Plateau. *Science China C Life Science,* **51**, 86-94.

Wang, C.T., Long, R.J., Wang, G.X., Liu, W., Wang, Q.L., Zhang, L. & Wu, P.F. (2010) Relationship between plant communities, characters, soil physical and chemical properties, and soil microbiology in alpine meadows. *Acta Prataculturae Sinica*, **19**, 25-34 (in Chinese).

Wang, G.J., Wang, S.P., Hao, Y.B. & Cai, X.C. (2005) Effect of grazing on the plant functional group diversity and community biomass and their relationship along a precipitation gradient in Inner Mongolia Steppe. *Acta Ecologica Sinica*, **25**, 1649-1656 (in Chinese).

Wang, J. & Baoyin, T. (2014) Species composition and the aboveground biomass of *Stipa glareosa* community in desert steppe. *Chinese Journal of Grassland,* **36**, 108-111 (in Chinese).

Wang, J.L. (2010) Preliminary research of plant and soil carbon sequestration potential in alpine meadow of Qinghai province [D]. *Qinghai: Northwest Institute of Plateau Biology, CAS* (in Chinese).

Wang, J.L., Ouyang, H., Wang, Z.H., Chang, T.J., Li, P., Shen, Z.X. & Zhong, Z.M. (2009) Influential factors and distribution characteristics of topsoil labile organic carbon in alpine grassland ecosystem at the south slope of Gongga south mountain-Laguigangri Mountain. *Acta Ecologica Sinica,* **29**, 3501-3508 (in Chinese).

Wang, K.B., Li, J.P. & Shangguan, Z.P. (2012) Biomass components and environmental controls in Ningxia grasslands. *Journal of Integrative Agriculture,* **11**, 2079-2087.

Wang, M., Su, Y.Z. & Yang, X. (2014) Spatial distribution of soil organic carbon and its influencing factors in desert grasslands of the Hexi Corridor, Northwest China. *PLoS One*, **9**, 1-8.

Wang, Q., Zhang, L., Li, L., Bai, Y., cao, J. & Han, X. (2009) Changes in carbon and nitrogen of Chernozem soil along a cultivation chronosequence in a semi-arid grassland. *European Journal of Soil Science*, **60**, 916-923.

Wang, Q.F., Chen, Y.M., Cao, Y., Cui, J. & Zhang, T. (2014) Topsoil carbon sequestration characteristics and influencing factors for two grasslands in Loess Hilly region. *Bulletin of Soil and Water Conservation*, **34**, 58-64 (in Chinese).

Wang, Q.J., Li, S.X., Wang, W.Y. & Jing, Z.C. (2008) The despondences of carbon and nitrogen reserves in plants and soils to vegetations cover change on *Kobresia pygmaea* meadow of Yellow River and Yangtze River source region. *Acta Ecologica Sinica,* **28**, 885-894 (in Chinese).

Wang, X.L., Wang, Y., Shi, H.H., Liu, Z.Y. & Ma, D.M. (2014) Soil organic carbon density under different land use types on the Nanchangshan Island of Miaodao Archipelago. *Acta Scientiae Circumstantiae,* **34**, 1009-1015 (in Chinese).

Wang, X.X., Dong, S.K., Yang, B., Li, Y.Y. & Su, X.K. (2014) The effects of grassland degradation on plant diversity, primary productivity, and soil fertility in the alpine region of Asia's headwaters. *Environmontal Monitoring and Assessment,* **186**, 6903-6917.

Wang, Y. (2012) The study of characteristicis of soil organic carbon and aggregates in different degraded alpine meadow. *Jiangsu: Nanjing Agricultural University* (in Chinese).

Wang, Z., Luo, T.X., Li, R.C., Tang, Y.H. & Du, M.Y. (2013) Causes for the unimodal pattern of biomass and productivity in alpine grasslands along a large altitudinal gradient in semi-arid regions. *Journal of Vegetation Science,* **24**, 189-201.

Wang, Z., Yun, X.j., Wei, Z.J., Schellenberg, M.P., Wang, Y.F., Yang, X. & Hou, X.Y. (2014) Responses of plant community and soil properties to inter-annual precipitation variability and grazing durations in a desert steppe in Inner Mongolia. *Journal of Integrative Agriculture,* **13**, 1171-1182.

Wei, W.D. & Liu, Y.H. (2014) Characteristics analysis of soil microbial biomass carbon on degradated alpine grassland. *Acta Agriculturae Boreali-occidentalis Sinica,* **23**, 205-210 (in Chinese).

Wei, X.R., Shao, M.A., Fu, X.L., Horton, R., Li, Y. & Zhang, X.C. (2009) Distribution of soil organic C, N and P in three adjacent land use patterns in the northern Loess Plateau, China. *Biogeochemistry*, **96**, 149-162.

Wen, H.Y., Niu, D.C., Fu, H. & Kang, J. (2013) Experimental investigation on soil carbon, nitrogen, and their components under grazing and livestock exclusion in steppe and desert steppe grasslands, Northwestern China. *Environmental Earth Sciences,* **70**, 3131-3141.

Wen, J., Zhou, H.K., Yao, B.Q., Li, Y.K., Zhao, X.Q., Chen, Z., Lian, L.Y. & Guo, K.X. (2014) Characteristics of soil respiration in different degraded alpine grassland in the source region of Three-River. *Chinese Journal of Plant Ecology*, **38**, 209-218 (in Chinese).

Wen, L., Dong, S.K., Li, Y.Y., Wang, X.X., Li, X.Y., Shi, J.J. & Dong, Q.M. (2012) The impact of land degradation on the C pools in alpine grasslands of the Qinghai-Tibet Plateau. *Plant and Soil,* **368**, 329-340.

Wu, G.L., Ren, G.H., Dong, Q.M., Shi, J.J. & Wang, Y.L. (2014) Above- and belowground response along degradation gradient in an alpine grassland of the Qinghai-Tibetan plateau. *Clean-Soil Air Water*, **42**, 319-323.

Wu, J.S., Zhang, X.Z., Shen, Z.X., Shi, P.L., Yu, C.Q. & Chen, B.X. (2014) Effects of livestock exclusion and climate change on aboveground biomass accumulation in alpine pastures across the Northern Tibetan Plateau. *Chinese Science Bulletin*, **59**, 4332-4340.

Wu, L. & Zhang, X.S. (2006) Characters of forage resources and the development of pastoral industry in the farming-pastoral zone of the Songnen Plain. *Acta Ecologica Sinica*, **26**, 601-609 (in Chinese).

Wu, L., He, N., Wang, Y. & Han, X. (2008) Storage and dynamics of carbon and nitrogen in soil after grazing exclusion in grasslands of Northern China. *Journal of Environment Quality,* **37**, 663-668.

Wu, X., Li, Z.S., Fu, B.J., Lu, F., Wang, D.B., Liu, H.F. & Liu, G.H. (2014) Effects of grazing exclusion on soil carbon and nitrogen storage in semi-arid grassland in Inner Mongolia, China. *Chinese Geographical Science,* **24**, 479-487.

Xiao, C.W., Janssens, I.A., Liu, P., Zhou, Z.Y. & Sun, O.J. (2007) Irrigation and enhanced soil carbon input effects on below-ground carbon cycling in semiarid temperate grasslands. *New Phytologist,* **174**, 835-846.

Xiao, X.P., Song, N.P., Xie, T.T. & Fang, K. (2013) Formation mechanism and community characteristics of fenced grassland in desert steppe. *Acta Prataculturae Sinica,* **22**, 321-327 (in Chinese).

Xiong, D.P., Shi, P.L., Sun, Y.L., Wu, J.S. & Zhang, X.Z. (2014) Effects of grazing exclusion on plant productivity and soil carbon, nitrogen storage in alpine meadows in northern Tibet, China. *Chinese Geographical Science,* **24**, 488-498.

Xiong, X.G. & Han, X.G. (2006) Dynamics of the small-scale heterogeneity of the soil carbon and nitrogen resources associated with *Caragana microphylla* in Inner Mongolia degraded steppe. *Acta Ecologica Sinica*, **26**, 483-488 (in Chinese).

Xue, X.J. (2009) Response of simulated climate change on the changes of soil and plant carbon and nitrogen with different altitude in alpine meadow [D]. *Qinghai: Northwest institute of plateau biology, CAS* (in Chinese).

Yan, Y., Liu, S.Z. & Zhou, W. (2006) Dynamic of grassland biomass in different degenerative stages. *Wuhan University Journal of Natural Sciences*, **11**, 958-962.

Yan, Y., Zhang, J.G., Zhang, J.H., Fan, J.R. & Li, H.X. (2005) The belowground biomass in alpine grassland in Nakchu Prefecture of Tibet. *Acta Ecologica Sinica*, **25**, 2819-2823 (in Chinese).

Yang, H.T., Li, X.R., Wang, Z.R., Jia, R.L., Liu, L.C., Chen, Y.L., Wei, Y.P., Gao, Y.H. & Li, G. (2014) Carbon sequestration capacity of shifting sand dune after establishing new vegetation in the Tengger Desert, northern China. *The Science of the total environment,* **478**, 1-11.

Yang, J.P., Mi, R. & Liu, J.F. (2008) Variations in soil properties and their effect on subsurface biomass distribution in four alpine meadows of the hinterland of the Tibetan Plateau of China. *Environmental Geology,* **57**, 1881-1891.

Yang, X.X., Ren, F., Zhou, H.K. & He, J.S. (2014) Responses of plant community biomass to nitrogen and phosphorus additions in an alpine meadow on the Qinghai-Xizang Plateau. *Chinese Journal of Plant Ecology,* **38**, 159-166 (in Chinese).

Yang, Y., Niu, D.C., Wen, H.Y., Zhang, B.L., Dong, Q., Chen, J.L. & Fu, H. (2012) Responses of soil particulate organic carbon and nitrogen along an altitudinal gradient on the Helan Mountain, Inner Mongolia. *Acta Prataculture Sinica*, **21**, 54-60(in Chinese).

Yang, Y., Guo, K., Zhao, L.Q., Zhao, H.W., Qiao, X.G., Liu, H.M. & Liu, C.C. (2014) Community characteristics of *Stipa roborowskyi* steppe in Xizang. *Chinese Journal of Plant Ecology,* **38**, 635-639 (in Chinese).

Yang, Y.H., Fang, J.Y., Ma, W.H., Guo, D.L. & Mohammat, A. (2010) Large-scale pattern of biomass partitioning across China's grasslands. *Global Ecology and Biogeography,* **19**, 268-277.

Yang, Y.H., Fang, J.Y., Ma, W.H., Smith, P., Mohammat, A., Wang, S.P. & Wang, W. (2010) Soil carbon stock and its changes in northern China's grasslands from 1980s to 2000s. *Global Change Biology,* **16**, 3036-3047.

Yu, P.J., Li, Q., Jia, H.T., Zheng, W., Wang, M.L. & Zhou, D.W. (2013) Carbon stocks and storage potential as affected by vegetation in the Songnen grassland of northeast China. *Quaternary International*, **306**, 114-120.

Zhang, F., Qi, B., Wen, F., Zhang, D.G., Wu, H. & Zhang, L. (2011) Analysis of the change of carbon storage in alpine arid grassland. *Acta Prataculturae Sinica,* **20**, 11-18 (in Chinese).

Zhang, J., Zhang, Q., He, J.M. & Sun, B. (2006) Estimation and analysis of grass biomass in desert by remote sensing. *Acta Ecologica Sinica,* **27**, 2294-2301 (in Chinese).

Zhang, J.H., Li, F.C., Wang, Y. & Xiong, D.H. (2014) Soil organic carbon stock and distribution in cultivated land converted to grassland in a subtropical region of China. *Environmental Management,* **53**, 274-283.

Zhang, J.Y., Wang, Y., Zhao, X., Xie, G. & Zhang, T. (2005) Grassland recovery by protection from grazing in a semi‐arid sandy region of northern China. *New Zealand Journal of Agricultural Research,* **48**, 277-284.

Zhang, T.H., Su, Y.Z., Cui, J.Y., Zhang, Z.H. & Chang, X.X. (2006) A Leguminous shrub (Caragana microphylla) in semiarid sandy soils of North China. *Pedosphere,* **16**, 319-325.

Zhang, X.H., Zhu, J.Z., Sun, Z.J., Jin, G.L., Zheng, W. & Gu, W.R. (2014) Influence of grazing intensity on the aboveground biomass and nutrient dynamics of community. *Pratacultural Science,* **31**, 116-124(in Chinese).

Zhao, C.Z. & Ren, H. (2012) Individual spatial pattern and spatial association of *Stipa krylovii* population in Alpine Degraded Grassland. *Acta Ecologica Sinica,* **32**, 6946-6954 (in Chinese).

Zhao, H.L., He, Y.H., Zhou, R.L., Su, Y.Z., Li, Y.Q. & Drake, S. (2009) Effects of desertification on soil organic C and N content in sandy farmland and grassland of Inner Mongolia. *Catena,* **77**, 187-191.

Zhao, H.T. (2007) Controls of carbon stocks and carbon mineralization potential in Inner Mongolia [D]. *Beijing: The Institute of Botany, CAS* (in Chinese).

Zhao, J.M. (2006) Study on the soil organic carbon stocks of alpine grassland under different degraded degrees in Eastern Qilian Mountains[D]. *Gansu: Gansu Agricutural University* (in Chinese).

Zhao, N., Zhuang, Y. & Zhao, J. (2014) Effects of grassland managements on soil organic carbon and microbial biomass carbon. *Pratacultural Science,* **31**, 367-374 (in Chinese).

Zhao, N.N., Guggenberger, G., Shibistova, O., Thao, D.T., Shi, W.J. & Li, X.G. (2014) Aspect-vegetation complex effects on biochemical characteristics and decomposability of soil organic carbon on the eastern Qinghai-Tibetan Plateau. *Plant and Soil,* **384**, 289-301.

Zhao, W., Chen, S.P., Han, X.G. & Lin, G.H. (2008) Effects of long-term grazing on the morphological and functional traits of Leymus chinensis in the semiarid grassland of Inner Mongolia, China. *Ecological Research,* **24**, 99-108.

Zheng, X.X., Zhao, J.M., Zhang, Y.G., Wu, Y.Q., Jin, T.T. & Liu, G.H. (2007) Variation of grassland biomass and its relationships with environmental factors in Hulunbeier, Inner Mongolia. *Chinese Journal of Ecology*, **26**, 533-538 (in Chinese).

Zhou, H.K., Tang, Y.H., Zhao, X.Q. & Zhou, L. (2006) Long-term grazing alters species composition and biomass of a shrub meadow on the Qinghai-Tibet Plateau. *Pakistan Journal of Botany,* **38**, 1055-1069.

Zhou, Y.T., Fu, G., Shen, Z.X., Zhang, X.Z., Wu, J.S., Li, Y.L. & Yang, P.W. (2013) Estimation model of aboveground biomass in the Northern Tibet Plateau based on remote sensing date. *Acta Prataculture Sinica*, **22**, 120-129(in Chinese).

Zhu, J.X., Wang, Q.F., He, N.P., Wang, R.M. & Dai, J.Z. (2013) Soil nitrogen mineralization and associated temperature sensitivity of different Inner Mongolian grasslands. *Acta Ecologica Sinica*, **33**, 6320-6327 (in Chinese).

Zhu, T.H., Cheng, S.L., Fang, H.J., Yu, G.R., Zheng, J.J. & Li, Y.N. (2011) Early responses of soil CO_2_ emission to simulating atmospheric nitrogen deposition in an alpine meadow on the Qinghai Tibetan Plateau. *Acta Ecologica Sinica*, **31**, 2687-2696 (in Chinese).

Zi, C.Y., Dan, W.X., Yang, L.X., Yan, Y. & Hui, F.J. (2013) Soil organic carbon and nutrients along an alpine grassland transect across Northern Tibet. *Journal of Mountain Science,* **10**, 564-573.

Zou, C.J., Wang, K.Y., Wang, T.H. & Xu, W.D. (2006) Overgrazing and soil carbon dynamics in eastern Inner Mongolia of China. *Ecological Research*, **22**, 135-142.

Zuo, X.A., Zhao, H.L., Zhao, X.Y., Guo, Y.R., Yun, J.Y., Wang, S.K. & Miyasaka, T. (2008) Vegetation pattern variation, soil degradation and their relationship along a grassland desertification gradient in Horqin Sandy Land, northern China. *Environmental Geology,* **58**, 1227-1237.

**Part 2 Unpublished data**

Chen Quansheng supply above-ground biomass in 55 sampling sites

Huang Mei supply above-, below-ground biomass and SOC in 37 sampling sites

Hu Zhongmin supply above-ground biomass in 368 sampling sites

Wang Changhui supply above-ground biomass and SOC in 70 sampling sites

Li Jie supply above-, below-ground biomass in 13 sampling sites

Xue Jingyue supply above-ground biomass in 35 sampling sites

He Nianpeng supply above-ground biomass in 338 sampling sites and below-ground biomass in 69 sampling sites

**Supplementary Appx. S5**

Appx. S5 Grassland classification at different scales ^†^

| **5** **grassland typ****es** | **18 grassland types** | **32 grassland subtypes** |
| --- | --- | --- |
| Steppe | Temperate steppe  Temperate meadow steppe  Temperate desert steppe  Alpine steppe  Alpine meadow steppe  Alpine desert steppe | Plain-hilly steppe  Mountain steppe  Sandy steppe  Plain-hilly meadow steppe  Mountain meadow steppe  Sandy meadow steppe  Plain-hilly desert steppe  Mountain desert steppe  Sandy desert steppe  Alpine desert steppe  Alpine steppe  Alpine meadow steppe |
| Meadow | Alpine meadow  Lowland meadow  Mountain meadow | Alpine meadow  Alpine saline meadow  Alpine swamp meadow  Low wetland meadow  Lowland saline meadow  Tidal saline lowland meadow  Lowland swamp meadow  Mid-low alpine meadow  Sub-alpine meadow |
| Desert | Alpine desert  Temperate steppe desert  Temperate desert | Temperate steppe desert  Alpine desert  Gravel desert  Sandy desert  Salty soil desert |
| Shrub-tussock | Warm temperate tussock  Warm temperate shrub-tussock  Tropical tussock  Tropical shrub-tussock  Tropical dry shrub-tussock | Warm temperate tussock  Warm temperate shrub-tussock  Tropical tussock  Tropical shrub-tussock  Tropical dry shrub-tussock |
| *Swamp* | *Swamp* | *Swamp* |

^†^Based on Zhang ^32^, [DAHV and CISNR](#_ENREF_3) ^25^. Italic type indicates the grassland type was not included in this paper.
